# Geological significance of new zircon U–Pb geochronology and geochemistry: Niuxinshan intrusive complex, northern North China Craton

**DOI:** 10.1371/journal.pone.0213156

**Published:** 2019-03-06

**Authors:** Cheng-long Shi, Xiao-zhong Ding, Yan-xue Liu, Jian-zhong Hu, Yang Song

**Affiliations:** 1 Institute of Geology, Chinese Academy of Geological Sciences, Beijing, China; 2 School of Scientific Research, China University of Geosience, Beijing, China; 3 The Institute of Mineral Resources, Chinese Academy of Geological Sciences, Beijing, China; University of Geneva, SWITZERLAND

## Abstract

The Huajian gold deposit is one of the largest hydrothermal intrusion-related gold deposits in eastern Hebei Province, located in the northern margin of the North China Craton (NCC). The mineralization in this district displays a close spatial association with the shoshonitic Niuxinshan intrusive complex (NIC), which contributes to the characterization of the metallogeny associated with convergent margin magmatism. In the current study, new geochronological and geochemical data are combined with previously published isotopic data, obtained from the granitic rocks in the NIC, to constrain the timing of the district’s tectonic setting transformation and determine its bearing on regional metallogeny. The new geochronological data constrain the timing of the tectonic transformation between 155 and 185 Ma. The NIC’s granitic rocks can be geochemically subdivided into two groups. One group’s geochemical signature exhibits steep rare earth element (REE) patterns with negligible Eu anomalies, lower Yb, higher Sr, and negative Nb–Ta–Ti (NTT) anomalies, which indicate a volcanic-arc environment with a thickened crust in a convergent setting. The other group exhibits flat REE patterns with obvious negative Eu anomalies, higher Yb, lower Sr, and weak NTT anomalies, which indicate an intra-plate extensional environment with a thinning crust. Combining geochronologic and isotopic data, the mineralization is Late Jurassic (~155 Ma). This is interpreted to be genetically related to the crystallization of the shallow crustal-sourced portions of this complex. Additionally, a tectonic model is presented that provides a plausible explanation for the abundant polymetallic mineralization that occurs in the northern margin of the NCC after 155 Ma.

## Introduction

**Fig 1:**
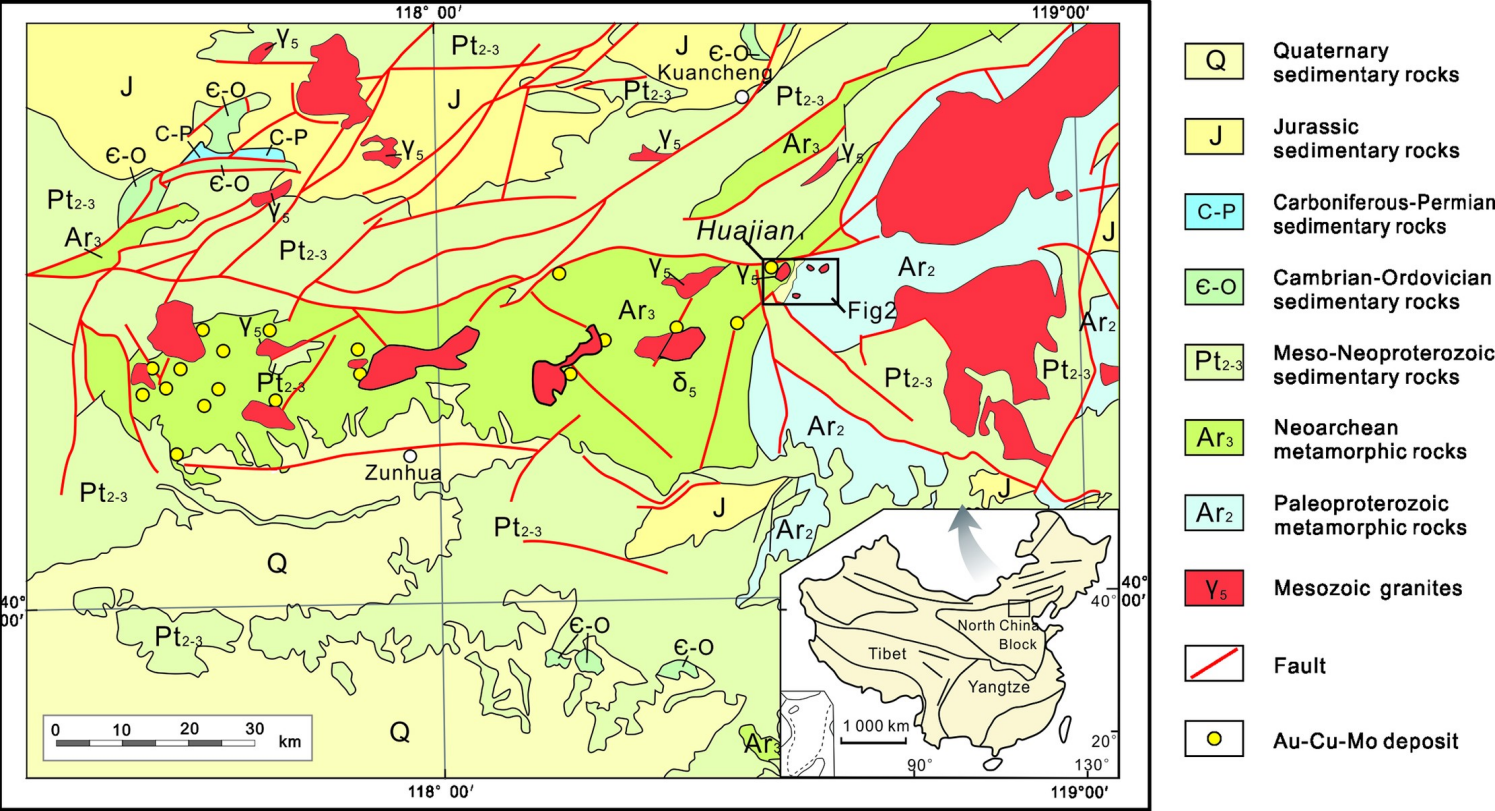
Regional geological sketch map of the northern part of the North China Craton (modified after[28]).

The association of mineralization with shoshonitic magmatism is a characteristic feature of the northern part of the North China Craton (NCC) [1–4]; however, this has not been previously described in detail for Huajian gold deposit (Fig 1).

Recently, dates for some deposits of the Middle Triassic-aged intrusive rocks, located in the northern part of the NCC, were obtained [5–12]. Particular tectonic environments are closely connected with the emergence and distribution of these deposits [5] [6–10], [11, 12], [13]. Therefore, the identification of varying tectonic settings in an area is useful for locating mineral deposits, also for identifying different metallogenic belts and their metallogenetic potential [14, 15].

These deposits are distributed along belts in the northern margin of the NCC and have a close spatial association with shoshonitic intrusions. The formation of these granitic rocks is due to the collision of northern NCC with a continental orogenic belt at the end of the Permian, which occurred during the final closure of the Paleo-Asian Ocean [2, 3, 16–19]. However, the tectonic regime transitioned after the Triassic, it changed from a volcanic arc to an intra-plate setting [20–23]. Subduction of the paleo-Pacific plate beneath the NCC altered the stress from NS- to EW-trending, and the lithosphere transitioned from thickening to thinning. There is considerable debate surrounding (i) the time at which the tectonic setting started its transition and (ii) its effect on regional magmatism and metallogeny [16, 21, 22, 24–27].

The current study presents new zircon U–Pb age and bulk-rock geochemistry analyses of several granitic rocks that were collected from the Huajian gold deposit. The evaluation of these data along with the authors’ published H–O–S–Pb isotopic data[28, 29], from paragenous sulfide and quartz in the mineralization zone contributes to the study’s objectives of (i) developing the tectonic evolution of the eastern Hebei Province, (ii) constraining the timing of the tectonic regime’s transition in the northern margin of the NCC from a volcanic arc to an intra-plate setting, and (iii) determining the effect of the tectonic regime’s transition on magmatism and mineralization.

## Geological setting and deposit styles

### Geological setting

The Huajian gold deposit is hosted by a Mesozoic-aged volcanic-intrusive complex that forms a part of a larger orogenic belt that is related to the volcanic and associated intrusive rocks that are observed in the northern margin of the NCC (Fig 1). The NCC is a Precambrian block that is assumed to have stabilized ~1.85 Ga [30, 31]. The Mesozoic-aged volcanic-intrusive complexes are part of the three main components of the northern margin of the NCC. The other components are the basement of the NCC and the thick sedimentary cover. The basement of the NCC is divided into highly metamorphosed Neoarchean (Zunhua Group) and Paleoproterozoic (Qianxi Group) rocks. The thick sedimentary cover includes marine clastic and carbonate, platform sediments of Mesoproterozoic–Neoproterozoic age, and Cambrian–Ordovician and Middle Carboniferous-Permian and Jurassic fluvial and deltaic sediments [32–35]. The structural framework of east Hebei is characterized using the following four fault systems: (1) the EW-trending basement fractures, which played a key role in controlling the sedimentation and magmatism; (2) the NE-trending faults with strike lengths ranging from tens of meters to several kilometers, which controlled the Yanshanian magmatism; (3) the NNE- to N-trending faults with strike lengths ranging from hundreds of meters to several kilometers which controlled the Mesozoic magmatic activities and mineralization; and (4) the NW-trending faults developed during the Indosinian (Triassic)–Yanshanian (Jurassic–Cretaceous) period. The Nappe structures that are located in east Hebei generally trend in a NE–NNE direction and thrust from the SE to the NW [36–38] (Fig 1).

**Fig 2:**
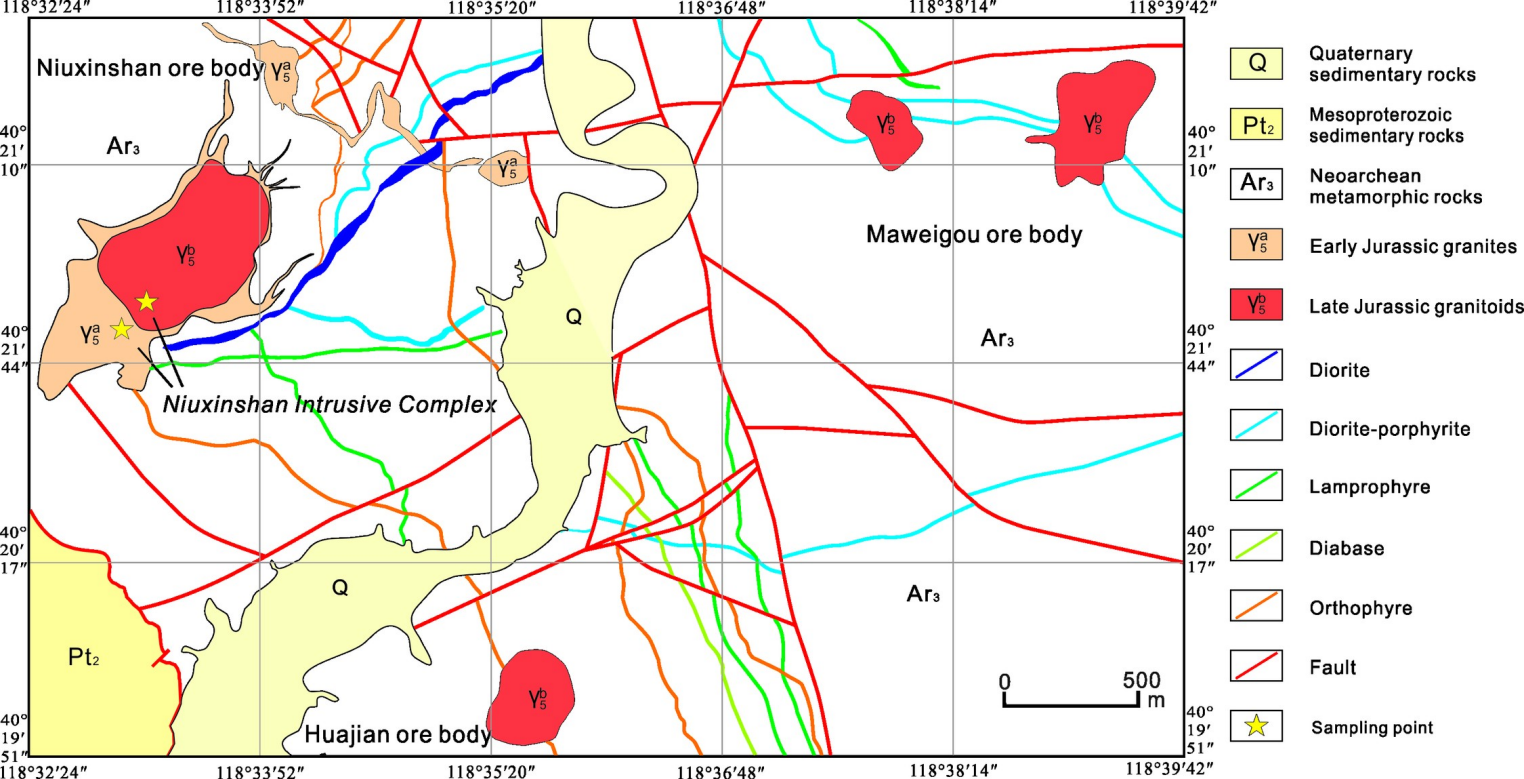
Geological sketch map of the Huajian gold deposit. (modified after[28]).

The Huajian gold deposit area is divided into three ore bodies that are as follows: the Niuxinshan, Huajian, and Maweigou ore bodies. Surface rocks in this area are dominated by the Zunhua Group, which mainly comprises a sequence of amphibolite, itabirite, and gneiss, including amphibolite, biotite plagioclase gneiss, hornblende gneiss, magnetite quartzite, and two-pyroxene granulite. The southwest portion of the ore deposit is hosted by Mesoproterozoic sedimentary rocks (Changcheng Group) that include the Dahongyu Formation (quartz sandstone and feldspathic quartz sandstone) and Gaoyuzhuang Formation (limestone and chert-nodule bearing dolomitic limestone) (Fig 2).

**Fig 3:**
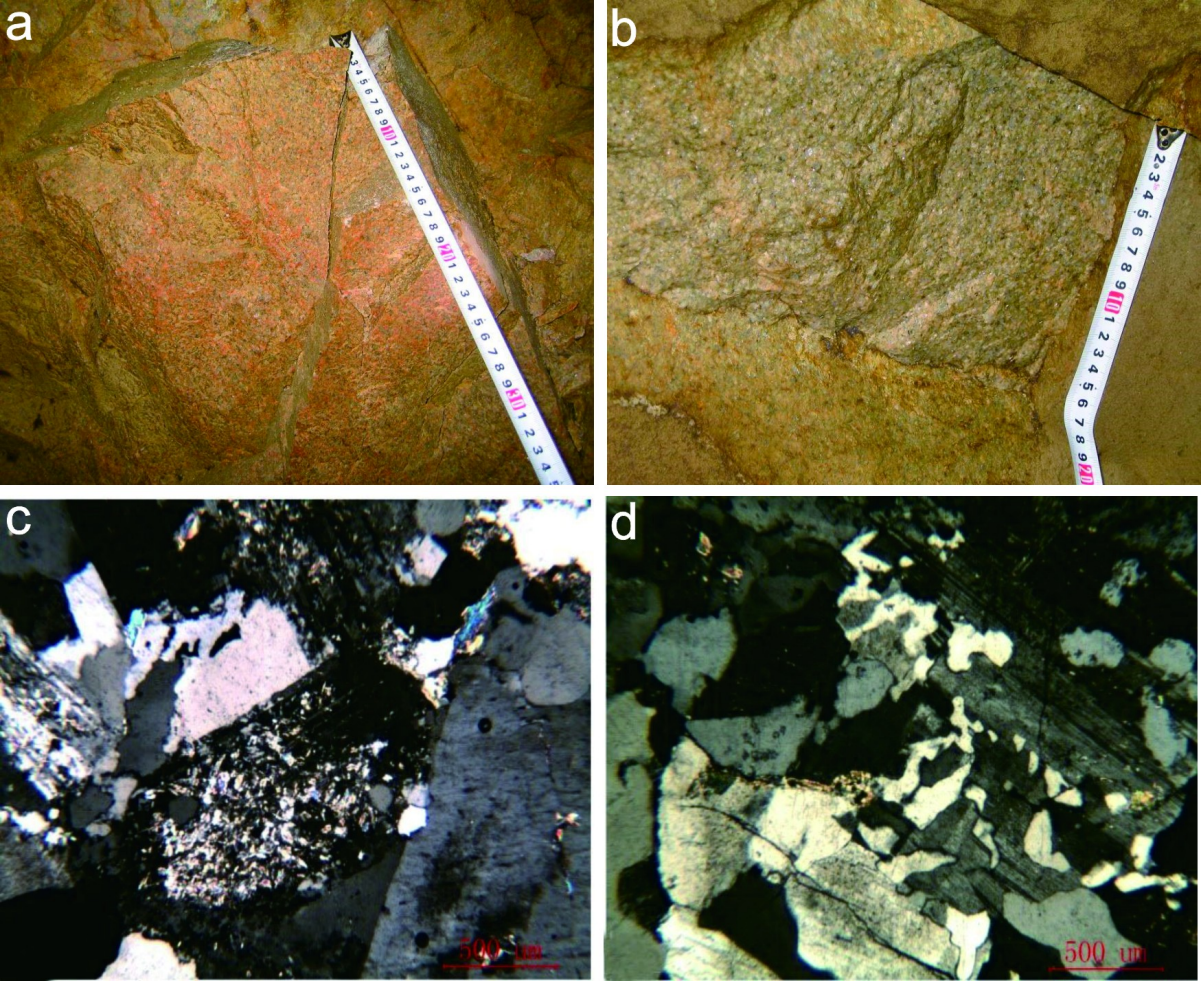
Representative photograph of NIC, the middle-coarse porphyritic granite (a, c) and the middle-fine grain granite (b, d).

The Niuxinshan intrusive complex (NIC) is a small (1 × 0.4 km) composite stock that intruded the Zunhua Group (Fig 2). It comprises two granitoid phases. The first-phase is coarse porphyritic granite located on the edge-phase of the NIC (Fig 3A and 3C). The remainder of the complex contains fine-grained granitic rocks (Fig 3B and 3D). The second-phase granitoid intruded into the center of the first-phase granite, and mineralization occurred in the central part of the NIC (Fig 3B and 3D). Swarms of diabase, diorite, diorite-porphyritic, orthophyre, and lamprophyre dykes that are located on the rim of the NIC’s main exposure are interpreted to be unrelated to the NIC, which indicates the presence of an extensive magma chamber at depth as well as different regional tectonic processes.

Emplacement of the NIC was probably facilitated and localized by the development of a major dilation zone, which contained regional faults that were oriented E-W, NNE and NNW; they constitute the complex structural pattern in this area. The NNE-trending faults are the main ore-forming structures to the northwest. The EW-trending faults control the central area, and the NNW-trending faults constrain the northeastern area. The prevailing tectonic stress that created the structures exerted effects during and after mineralization because they represent the predominant orientations of the mineralized quartz veins in the Niuxinshan, Huajian, and Maweigou ore bodies (Fig 2).

### Mineralization styles

Mineralization in this deposit comprises quartz vein-type and fracture-zone altered rock. The quartz vein-type gold mineralization mainly occurs in the gneissic rocks, which are extensively distributed throughout the ore blocks of Niuxinshan and Huajian. The quartz veins are controlled by fractures, which are oriented in various directions; however, they are observed to mainly trend NE and NNE. Mineralization has been discovered in more than 200 auriferous quartz veins over a length of 100–700 m with a width of 0.3–1.5 m. They form a broadly tabular envelope that dips 30°–45° to the northwest and strikes to the northeast. The veins are observed to often swell, pinch-out, and branch along the strike and dip. Majority of the veining is controlled by the NNE- and NW-trending fractures, and it occurs as parallel quartz veins, sheeted quartz veins, and quartz stockworks in the fracture zones.

The fracture-zone altered rock type is pervasively developed in the NIC and is distributed along the contact of the two intrusions. Gold is observed, especially, within the contact zone.

The ore mineralogy is relatively simple. Metallic minerals account for ~10% of the total minerals, whereas metal sulfides account for ~60%–70% of the metallic minerals. Pyrite is the main component of the metallic minerals; the others are galena, chalcopyrite, native gold, native silver, and electrum with occasional chalcocite, siderite, and scheelite. The gangue minerals are dominantly quartz, plagioclase, sericite (60–70 vol%), K-feldspar, chlorite, kaolinite, and calcite with minor fluorite and epidote. The texture of the ore is dominantly euhedral- to subhedral-granular and allotriomorphic-granular. The ore structure is banded, disseminated, and brecciated (Fig 3).

## Samples and analytical methods

Samples used for the Zircon U–Pb dating and geochemistry analyses were collected from the NIC located in the Huajian deposit (S1 and S2 Tables), China Geological Survey issued the permission for each location. Fig 2 depicts the distribution and the locations of the granitic rock samples. The detailed mineralogical characteristics of the analyzed samples have been presented in[28], and photomicrographs of the typical samples have been presented in [39]

### LA-ICP-MS U-Pb dating

Zircon U–Pb isotopes were dated using an inductively coupled plasma mass spectrometer (ICP-MS) (Neptune Plus multicollector; Thermo Scientific) at the Geological Lab Center, China University of Geosciences, Beijing. The zircons were separated using a magnetic separator and heavy liquids and were carefully selected based on their turbidity, color, shape, and size. Cathodoluminescence (CL) images were obtained using a microprobe microanalyzer (JXA-8100; JEOL Ltd.) at the Key Laboratory of Orogenic Belt and Crustal Evolution, Peking University. The data reduction, apparent age calculations, and isotopic ratios were processed using the ICPMS DataCal software [40]. The data processing used IsoplotEx 3 [41]. Secondary reference zircon of standard samples was using GJ-1 (~599 Ma) [42, 43]. The analytical procedure that was used to perform the analyses is described in detail in [44]

### Major- and trace-element analyses

Major and trace elements (including REE) were measured at the Geological Lab Center, China University of Geosciences, Beijing. The major elements were analyzed on fused-glass discs using inductively coupled plasma optical emission spectrometry (ICP-OES) equipped with Prodigy (Thermo Scientific iCAP 7000 Plus). The trace elements were analyzed using ICP-MS (Agilent 7500a) fitted with a 193 nm laser sampler. The sample preparation and analytical procedures are described in [45]. Monitor analyses followed the GSR-1 Chinese national standard[45]. Most of the major-element analytical errors were within 1%, except for P_2_O_5_ (5%), whereas those for the trace elements were within 10%.

## Results and discussion

### Results

#### Zircon U-Pb age

**Fig 4:**
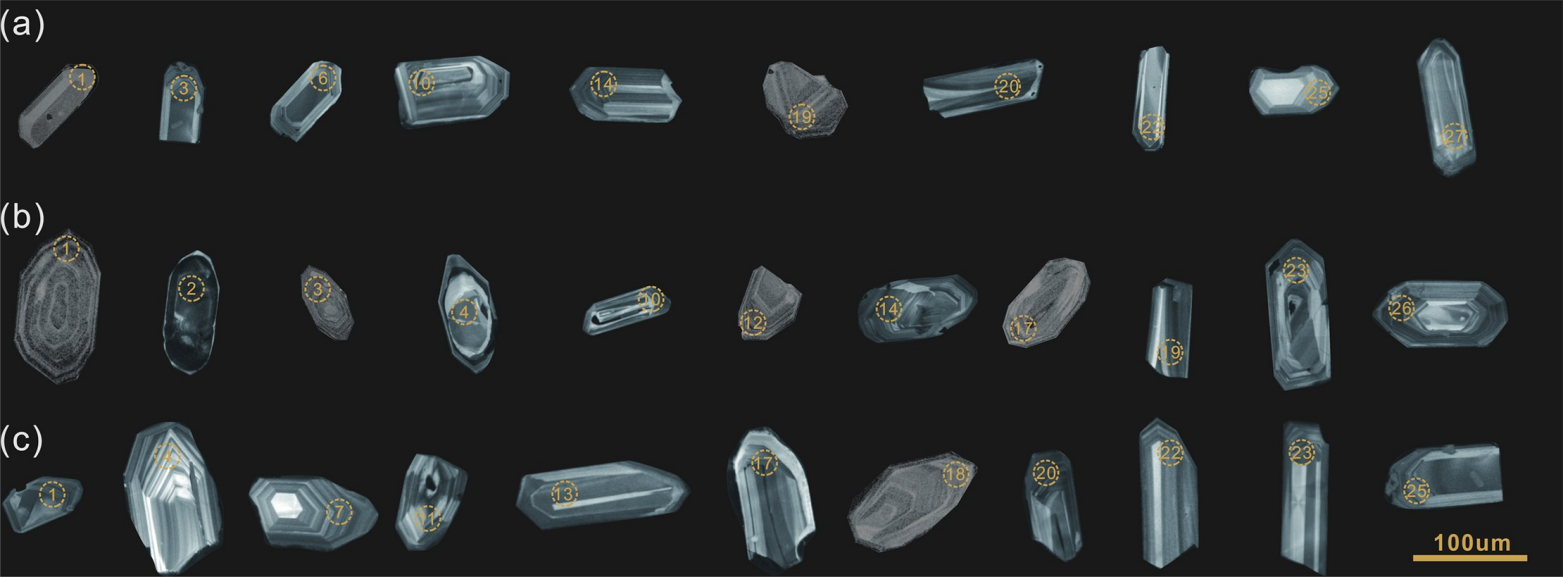
The cathodoluminescence (CL) images for zircons of Sample HNK103-4(a), HNK103-7(b), HNK103-16(c).

The results of LA-ICP-MS zircon U–Pb analyses are listed in S1 Table. All the data points were located on or close to the concordia, indicating minimal Pb-loss after zircon crystallization. The zircon grains were transparent to semi-transparent, colorless or light brown, and euhedral. They were generally 100–150 μm long with ~2:1–3:1 length to width ratios. The CL images indicated that most of the zircons contained no inherited cores and that they exhibited good oscillatory zoning (Fig 4). Many of the zircon analysis plot to the right of the Concordia line, this is due to the participation of the common Pb, but it is still within reasonable limits and has little effect on the ^206^Pb/^238^U age [46]

**Fig 5:**
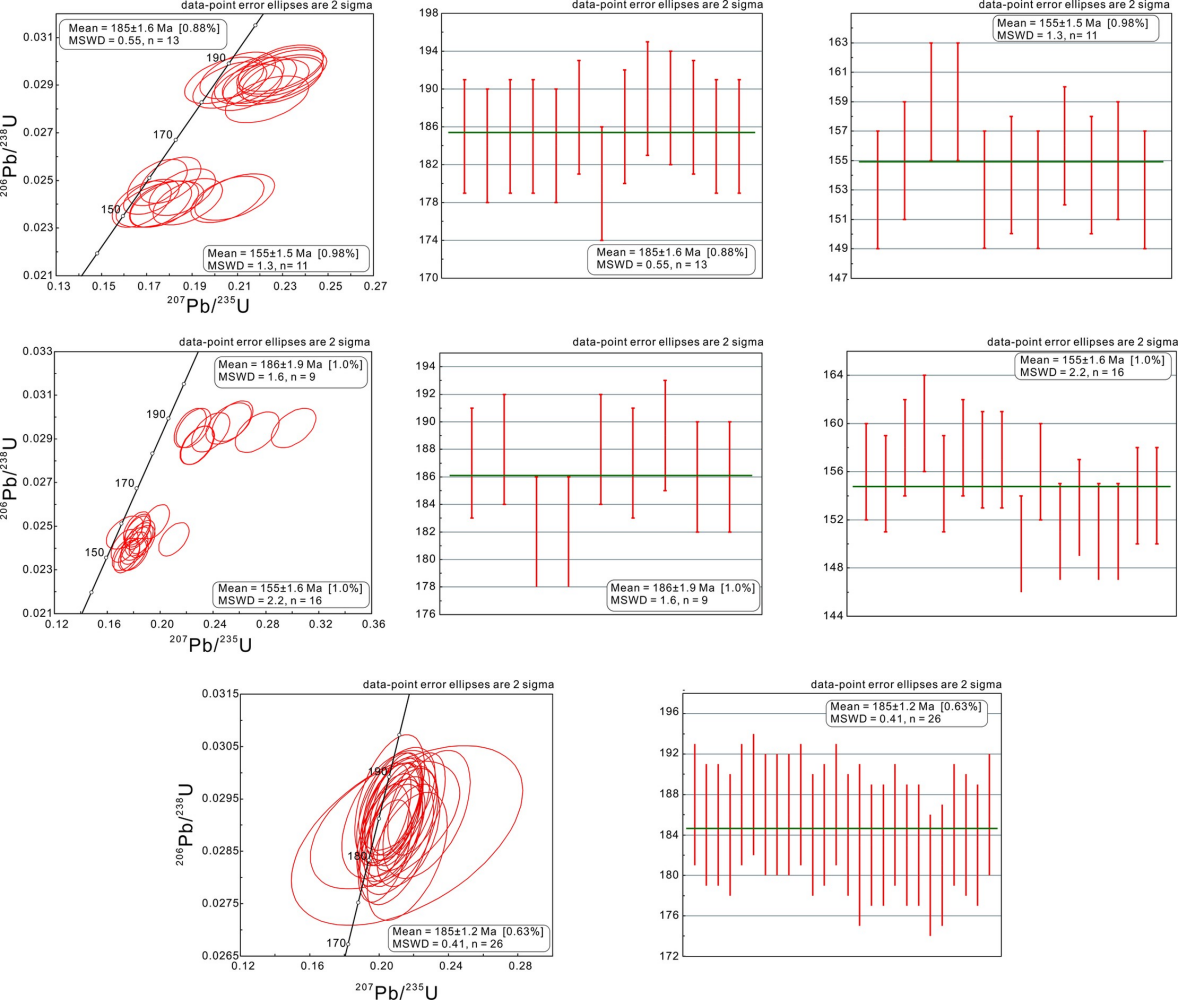
Representative zircon U–Pb concordia and diagrams of the samples’ weighted averages.

Twenty-four spots on 28 zircon grains were analyzed from HNK103-4, collected from the central part of the NIC. 13 inherited grains were observed; most of them were concordant (Fig 5). The weighted mean ^206^Pb/^238^U age of these 13 analyses was 185.4 ± 1.6 Ma (mean square of weighted deviates [MSWD] = 0.55, 95% confidence). The weighted mean ^206^Pb/^238^U age of the remaining 11 spots was 154.9 ± 1.5 Ma (MSWD = 0.55, 95% confidence) (Fig 5A–5C). The high Th/U ratios (ranging from 0.37 to 0.96), oscillatory zoning, and euhedral-shaped prisms indicated that they were magmatic zircons.

Twenty-seven spots on 28 zircons were analyzed from HNK103-7, collected from the central part of the NIC; most of these were reasonably concordant (Fig 5). The high Th/U ratios (ranging from 0.40 to 1.72), oscillatory zoning, and euhedral shape indicated a magmatic origin. Two of these spots (HS7-02 and HS7-04) were interpreted as zircons that were inherited from the surrounding rocks. The remaining spots generated a weighted mean of 186.1 ± 1.9 Ma for nine spots (MSWD = 1.6, 95% confidence) (Fig 5C) and 154.8 ± 1.6 Ma for 16 spots (MSWD = 2.2, 95% confidence) (Fig 5D–5F). These ages were very similar to those that were obtained from the HNK103-4 sample.

In the HNK103-16 sample, from the NIC’s margin, 29 spots on zircon grains were analyzed, and most of these were concordant (Fig 5). Excluding the disconcordant analyses (the ages of the inherited zircons (HS16-08, HS16-18, and HS16-27), the remaining 26 analyses exhibited a weighted mean ^206^Pb/^238^U age of 184.6 ± 1.2 Ma (MSWD = 0.41, 95% confidence) (Fig 5G and 5H). The high Th/U ratios and well-developed oscillatory zoning indicate that the grains are magmatic.

#### Major- and Trace-Element geochemistry

**Fig 6:**
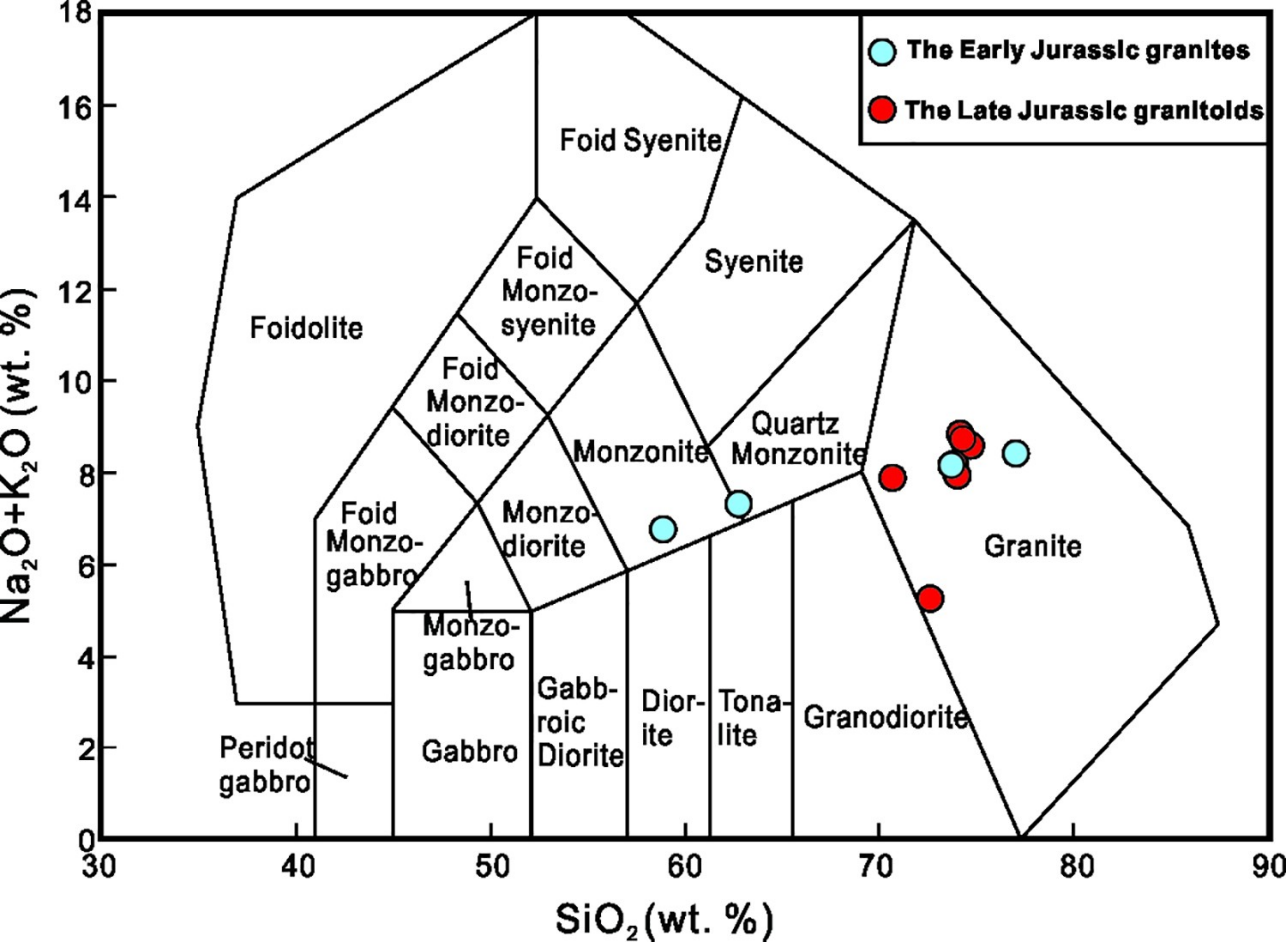
SiO_2_-(K_2_O+Na_2_O) (TAS) Diagram showing samples from the NIC (after [47]).

**Fig 7:**
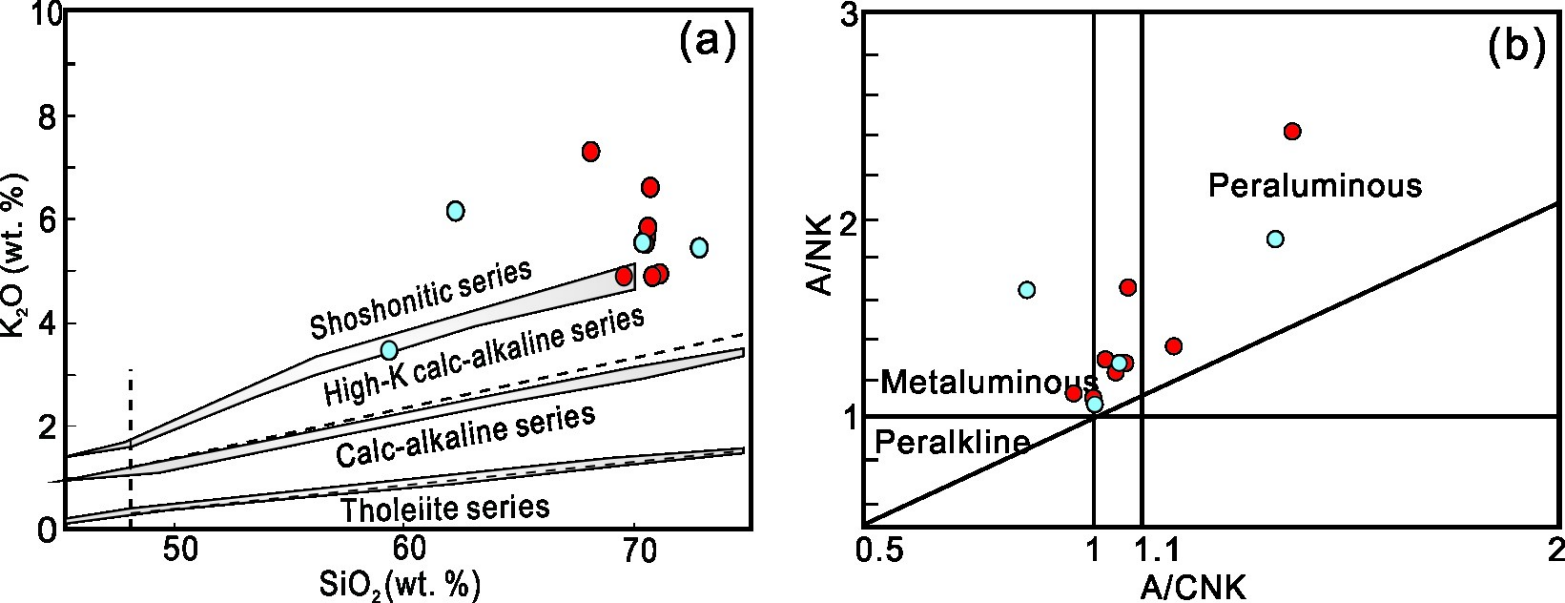
Plots of (a) SiO_2_ versus K_2_O (after Le Maitre et al., 1989, Rickwood et al., 1989); (b) A/NK versus A/CNK; A/NK = Al_2_O_3_/(Na_2_O + K_2_O), A/CNK = Al_2_O_3_/(CaO + Na_2_O + K_2_O), molecular ratio; (after [48]). Symbols are same as Fig 6.

Representative analyses of the NIC samples are presented in S2 Table. According to the classification by Peccerillo and Taylor (1976), the Early Jurassic granites on a total alkali-SiO_2_ (TAS) diagram, exhibit a well-defined trend moving from the granite to the quartz monzonite and monzonite fields (Fig 6) and belonged to the shoshonitic series (Fig 7). A molecular Al_2_O_3_/(Na_2_O + K_2_O) (A/NK) versus a molecular Al_2_O_3_/(CaO + Na_2_O + K_2_O) (A/CNK) diagram shows that all of the samples are peraluminous (A/CNK>1 and A/NK>1) except for one sample, which is metaluminous (A/CNK<1 and A/NK>1) (Fig 7). The Late Jurassic granites, on a TAS diagram, exhibit a well-defined trend through the granite field (Fig 6). The high total alkali to SiO_2_ ratio also defined the central NIC granites as shoshonitic (Fig 7).

**Fig 8:**
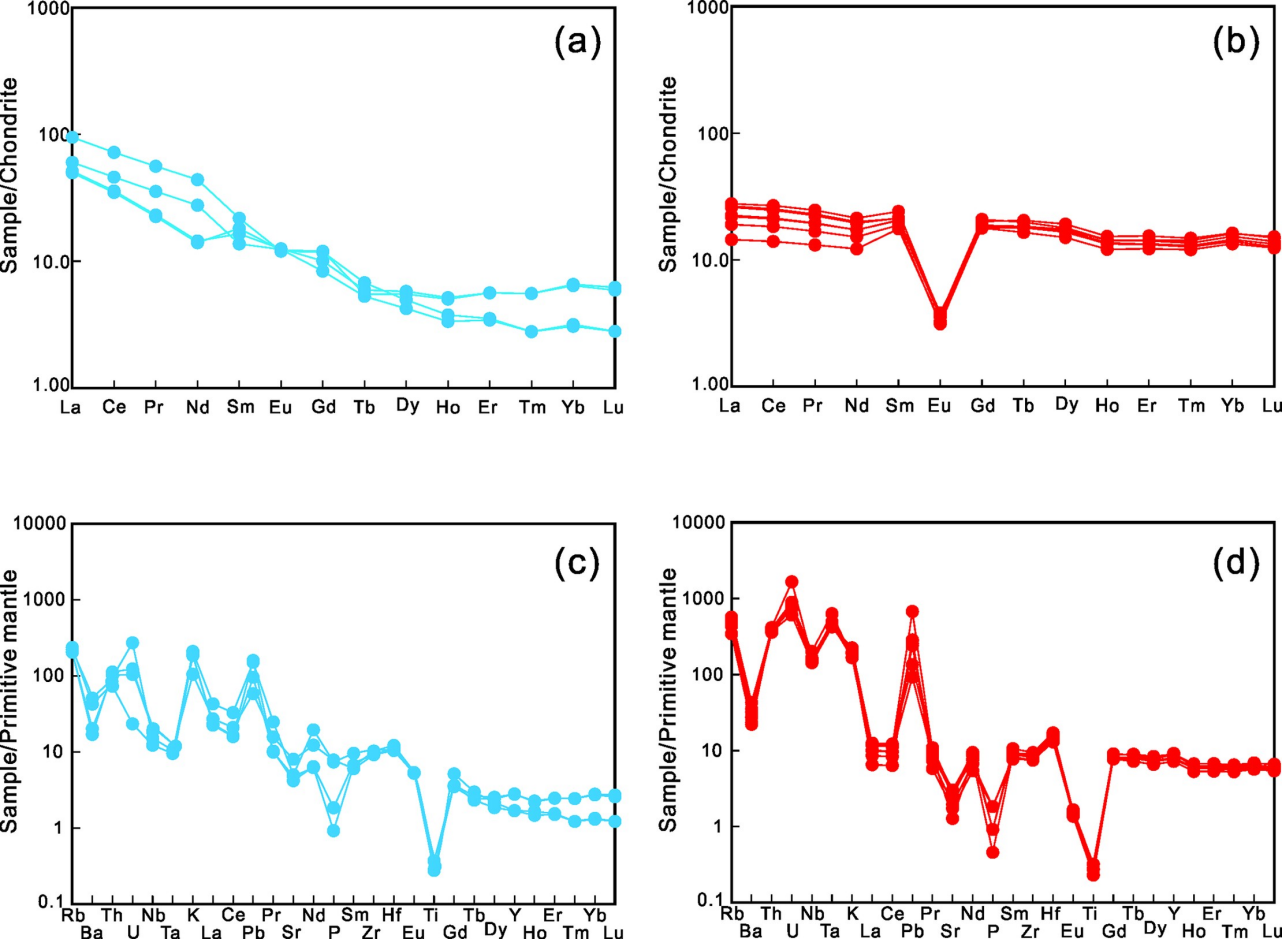
Chondrite-normalized REE and primitive mantle-normalized trace element patterns for Early Jurassic (a, c) and Late Jurassic (b, d) granitic rocks in the study area. Normalization values for chondrite and primitive mantle are from[49], and[50], respectively.

The Early Jurassic granitic rocks exhibit moderate REE content with negligible Eu anomalies; they are depleted in heavy rare earth elements (HREEs) and enriched in light rare earth elements (LREEs) (Fig 8A) with La/Lu ratios ranging from 9.50 to 17.97. The Early Jurassic granitic rocks are enriched in large-ion lithophile elements (LILEs), such as Cs, Rb, Ba, Th, U, and Pb; however, they are depleted of high field strength elements (HFSEs), depicting negative Ta, Nb, P, and Ti anomalies (Fig 8C).

The Late Jurassic granitic rocks exhibit relatively flat REE patterns with prominent negative Eu anomalies (0.16–0.21). They show a marked depletion of Ti; however, they are only weakly depleted in Ta and Nb as compared with the Early Jurassic rock samples (Fig 8B and 8D).

## Discussion

### Geochronologic feature of the Mesozoic granitic rocks of the NIC

The formation of NCC can be mostly explained by the evolving orogenic processes that span the Precambrian to Mesozoic eras, which were connected to ocean-formation, including subduction and closure, terrain patching, continental collision, and crustal extension [2, 3, 16, 18, 27, 51–58]. Numerous studies have suggested that the tectonic history of the northern margin of the NCC, especially from the Late Paleozoic to Early Mesozoic, includes the final closure of the Paleo-Asian Ocean [2–4, 17, 24, 59–63].

However, the tectonic regime transitioned after the Triassic in the northern margin of the NCC, and the tectonic history and geological setting of this area are still under debate [16, 20, 22, 23, 27]. Engebretson (1985) [64] proposed that, in Early Jurassic (180 Ma), the paleo-Pacific plate exhibited a velocity of 47 mm/a and began to be subducted relative to the NCC. In Late Jurassic (~145 Ma), the paleo-Pacific plate’s velocity increased rapidly to 300 mm/a, resulting in an increasing dip angle of the subducted plate. In the Early Cretaceous (~120 Ma), the subduction velocity slowed to ~207 mm/a. At ~60 Ma, the subduction of the paleo-Pacific plate was completed. Mao (2005) [21] suggested that large-scale mineralization in the northern margin of the NCC during the Mesozoic period occurred in the following three stages: (i) a post-collisional process at approximately 200–160 Ma, (ii) a tectonic transitional process at ~140 Ma, (iii) and a lithospheric thinning process at ~120 Ma.

**Fig 9:**
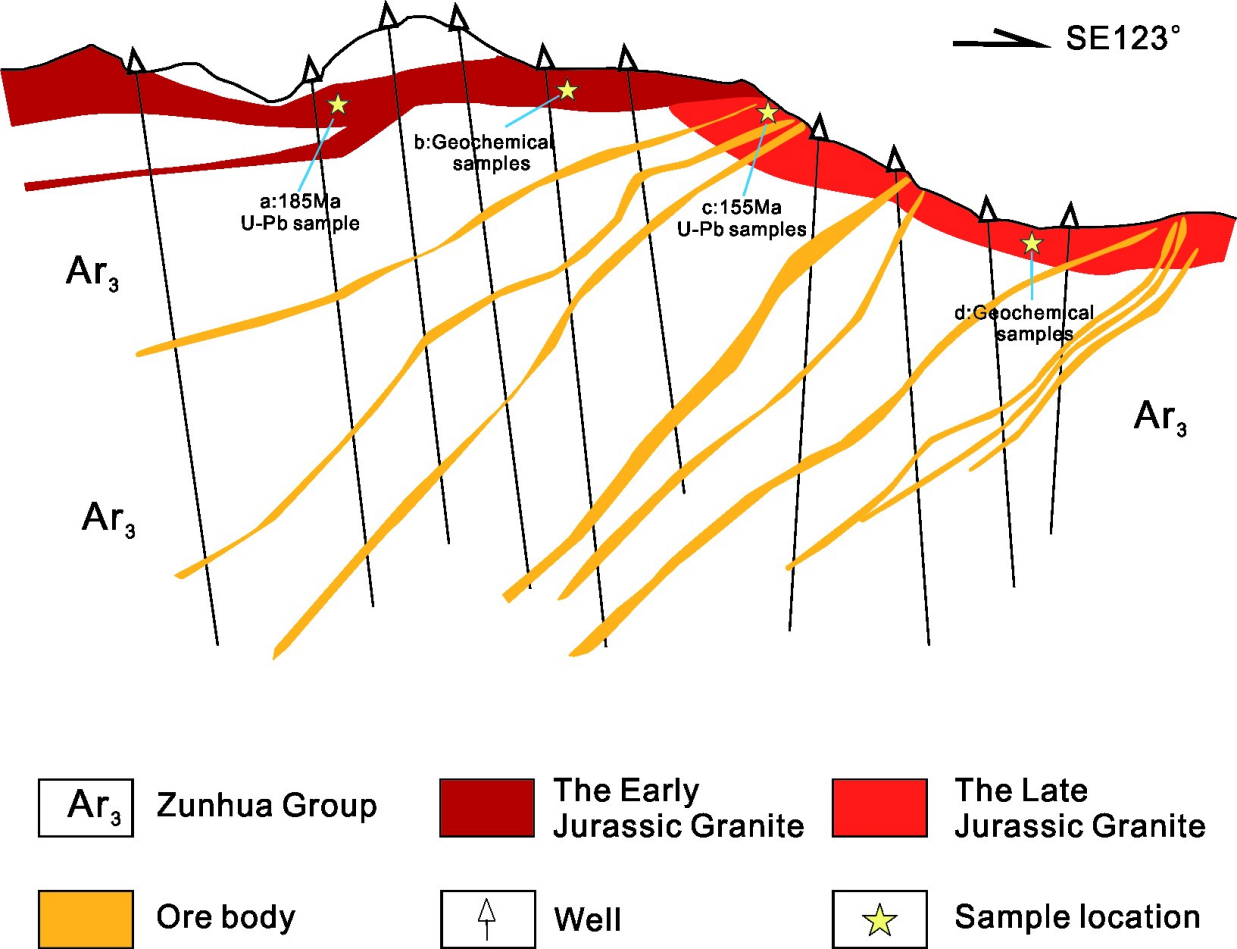
Profile of the mine structure in the Huajian deposit. Revised from [39].

In this study, for the samples HNK103-4 and HNK103-7, it contains zircon crystals with two age populations. This is because the sampling location for age samples from the second-phase granitoid was near the junction between the two phases of granitic rocks (Fig 9C); therefore, we collected a piece of the 155 Ma granite that had a xenolith from the 186 Ma granite within it. And then during crushing these two distinct samples became mixed. As a result, the mean age of 185 Ma, Early Jurassic, was interpreted to be the age of the first emplacement stage of the NIC. In addition, the geochemical samples from the second-phase granitoid are closer to the center part and away from the junction, so the two different features of two phases of granitic rocks do not interfere with each other (Fig 9D). The new zircon U–Pb dating of the NIC indicates that the Late Mesozoic magmatic events in the research region can be divided into two periods: (i) Early Jurassic (~185 Ma) with a volcanic-arc setting due to the initiation of the paleo-Pacific plate’s subduction and (ii) Late Jurassic (~155 Ma) with an intra-plate extensional setting due to the increasing velocity and dip angle of the subducting plate. Therefore, ~185 Ma is the probable timing of the tectonic regime beginning to enter the transition stage, whereas ~155 Ma is the feasible timing of metallogenesis. The reasons will be discussed in detail below.

### Geochemistry of the Mesozoic granitic rocks of the NIC

The Early Jurassic granites that intruded into the northern margin of the NIC exhibit steep HREE patterns and contain relatively low contents of Y and HREE (Fig 8A and 8C), indicating that garnet rather than amphibole was the residual mineral in the source region. The Early Jurassic granites exhibit slight or no obvious negative Eu anomalies along with relatively low contents of Sr (88.02–168.80 ppm) (Fig 8A and 8C); this indicates a stability field condition for plagioclase in the source rocks during melting. Patiño Douce (1995) [65] indicated that plagioclase maintains stability at a pressure of less than 15 kbar; as pressure increases to become greater than 12.5 kbar, garnet will be formed as the typical residual phase during the melting-dehydration process of the metasedimentary protoliths. If plagioclase and garnet both exist in the residual phase, this indicates that the granitic rocks’ source region was probably under pressures of 12.5–15 kbar and was relatively deep, ~40–50 km.

In contrast to the Early Jurassic granites, the Late Jurassic granites exhibit flat HREE patterns (Fig 8B and 8D) and displayed high contents of Yb, Y, and HREE (Fig 8B and 8D). These characteristics suggest that garnet was not a residual phase during melting in the source region [66–70]. Intensive negative Eu anomalies and relative depletion of Sr require the same stability field as required by plagioclase in the source rocks during melting, which can be achieved at relatively shallow levels in the crust (~20–30 km). Experimental petrology indicates that plagioclase is the main residual phase of hornblende-bearing granites during dehydration melting at a relatively shallow level of less than 30 km in the crust with a 20% to 40% fraction melt at a pressure of 4 kbar.

**Fig 10:**
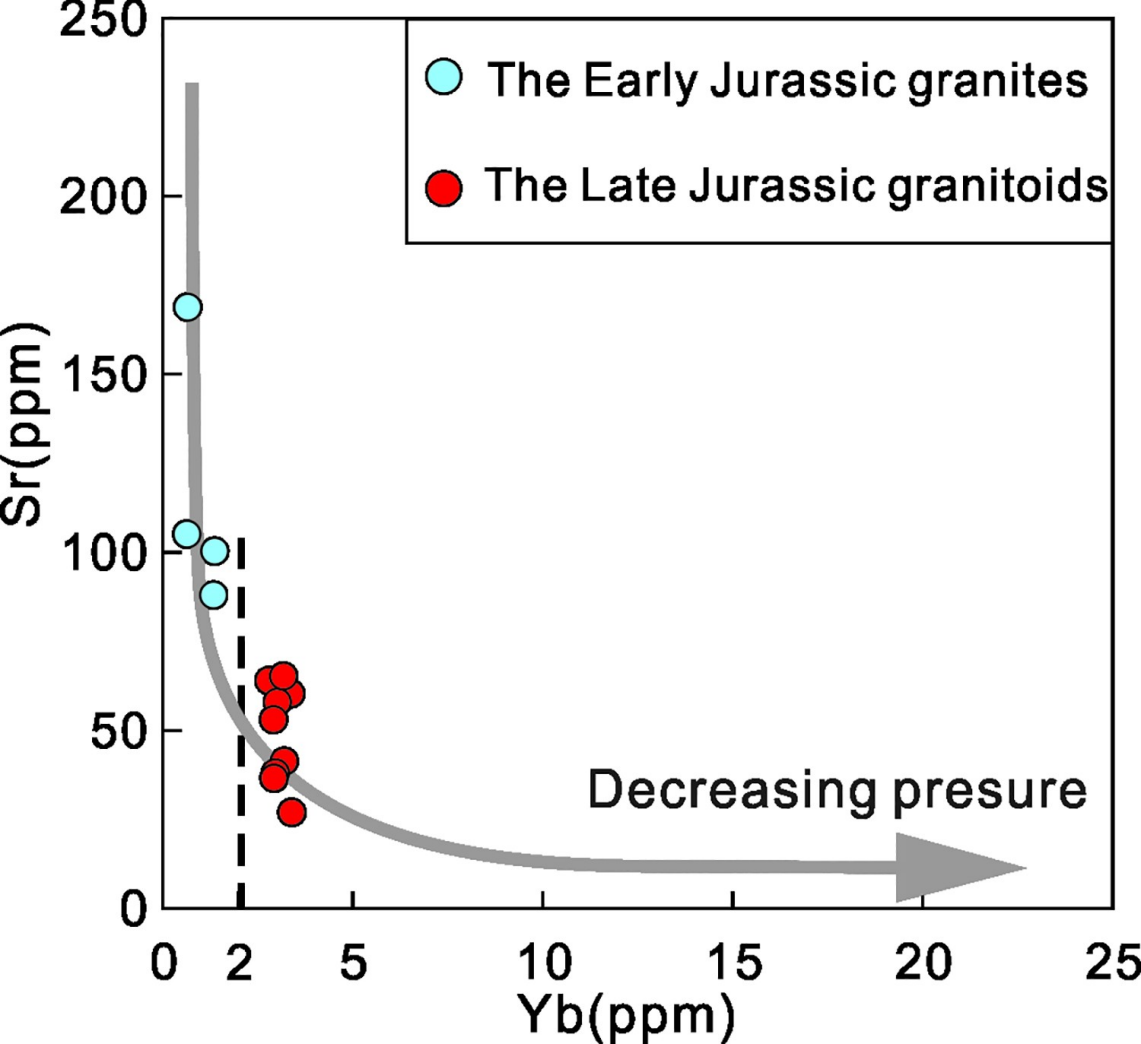
Sr vs Yb diagram for Jurassic granitic rocks in NIC[71].

As depicted in Fig 10, the granite samples from the NIC evolved from lower Yb-higher Sr to higher Yb-lower Sr during the Early to Late Jurassic. This indicates that the source region changed from deep to shallow and that the residual phase changed from garnet and plagioclase to only plagioclase. This evolution indicates a tectonic transition regime that changed from a compressional environment to an extensional environment that was accompanied by a thinning crust.

**Fig 11:**
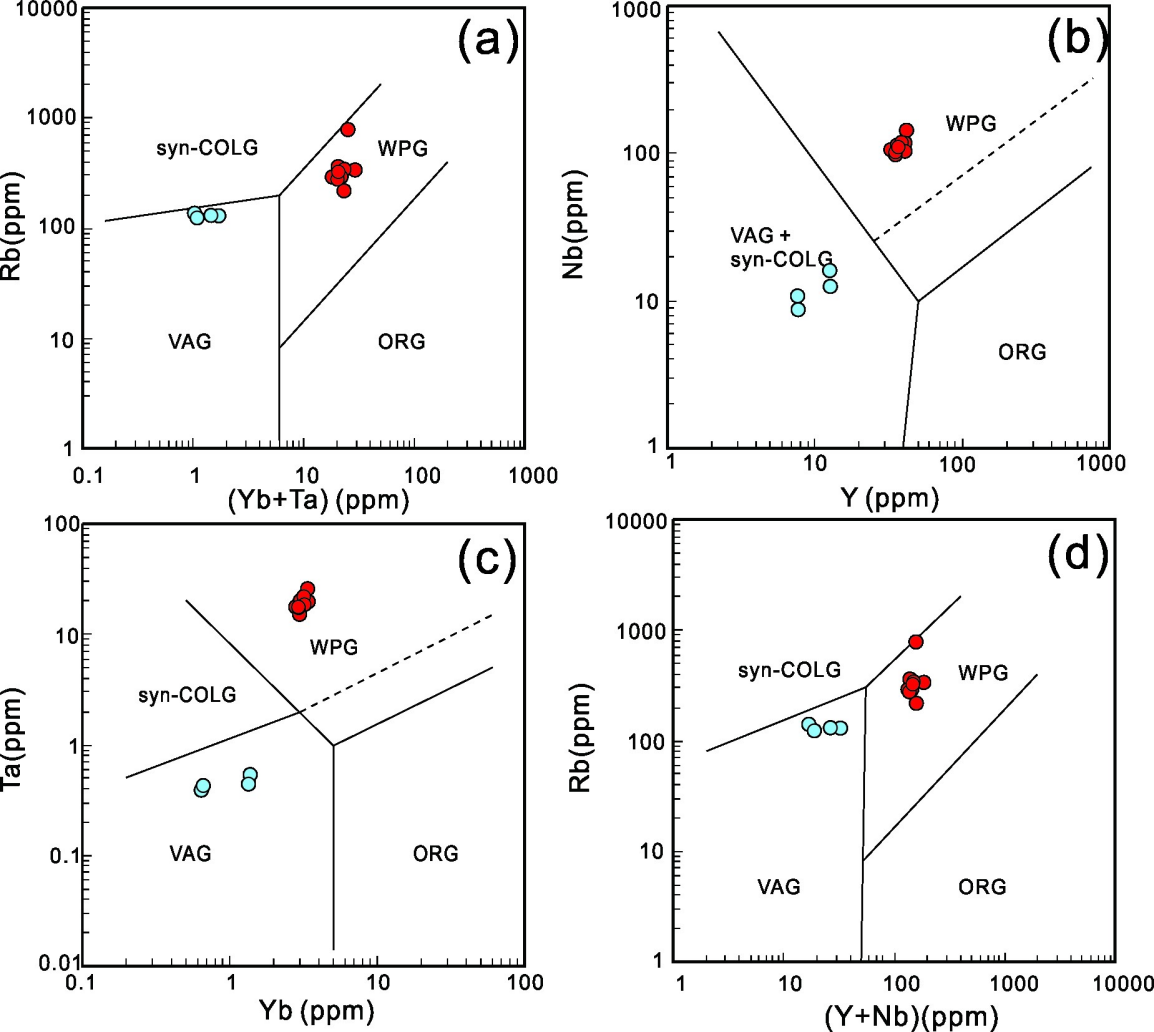
Trace element discrimination diagram of the tectonic setting after [72]. Rb vs. Yb+Ta,(a), Nb vs. Y (b), Ta vs. Yb (c), Rb vs. Y+Nb (d). VAG, volcanic-arc granites; ORG, ocean-ridge granites; WPG, within-plate granites; Syn-COLG, syn-collisional granites; Late or Post-COLG, late or syn-collisional granites. Symbols are same as Fig 6.

Various tectonic discrimination diagrams [72] have been used to discriminate the tectonic setting of the Early and Late Jurassic granitoid samples from the NIC. Early Jurassic rocks fall into the region of volcanic-arc granite in Rb vs. Yb + Ta, Ta vs. Yb, and Rb vs. Y + Nb diagrams (Fig 11A, 11C and 11D) and volcanic-arc granite and syn-collisional granite in Nb vs. Y diagrams (Fig 11B), whereas Late Jurassic granites fall into the region of within-plate granite in all the discrimination diagrams that are given below.

Additionally, the Early Jurassic granites depict negative NTT anomalies, referred to as the depletion of Nb, Ta, and Ti. Despite this being a typical indicator of a subduction setting, Ringwood (1990) indicated that such negative anomalies could also be produced by the retention of rutile during partial melting in source regions and fractional crystallization of rutile and/or titanite during magma evolution. However, the retention or fractional crystallization of rutile and titanite will not only cause the depletion of NTT and but also produce negative anomalies of Hf and Zr, and an increased ratio of Nb/Ta [73, 74]. This does not correspond with the obvious positive Hf and Zr anomalies and the high Hf and Zr contents that are reported in this study (Fig 8A).

Previous studies have suggested that, if basement rocks comprise protoliths connected by a paleo-arc, such as the magmatic rocks in the Zunhua Group, they will undergo remelting, and the NTT anomalies will be inherited[72, 75]. However, because the Zunhua Group represents only a series of the greenschist facies, 2–7-kbar low-grade metamorphic rocks, it is impossible to form igneous rocks with negligible Eu anomalies and REE patterns enriched of LREEs and depleted of HREEs by remelting, which requires pressures that are larger than 12.5 kbar. Therefore, subducted sediments or slab-derived fluids would more feasibly produce the negative NTT anomalies that are observed in NIC’s igneous rocks [76, 77].

**Fig 12:**
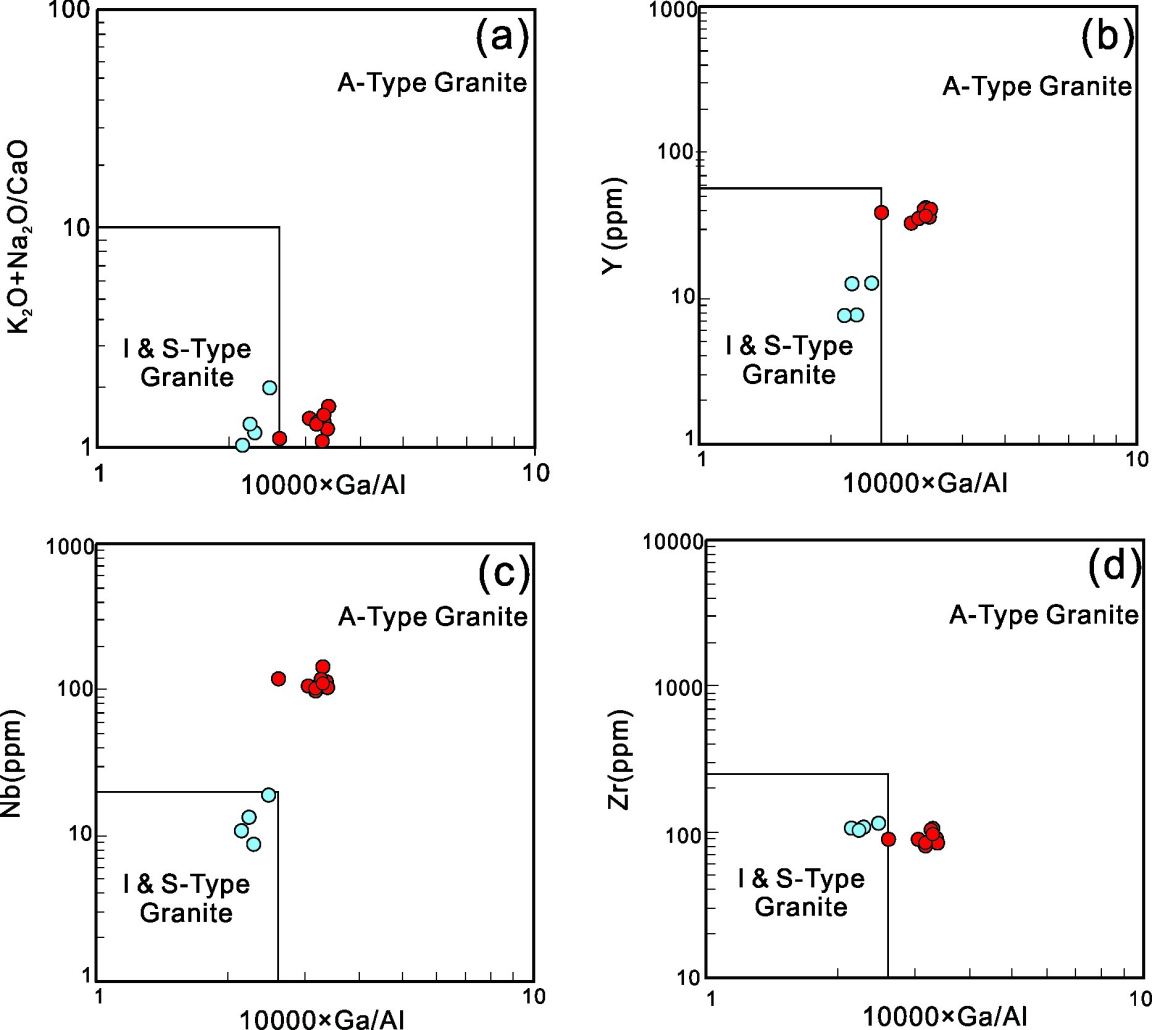
(K_2_O+Na_2_O) (a), Y (b), Nb (c), Zr (d)-10000 Ga/Al diagram(after [78]) of the granites from the NIC. Symbols are same as Fig 6.

In contrast, Late Jurassic granites only contain relatively weak negative NTT anomalies, which precludes an arc-related origin. However, they exhibit some characteristics of A-type granites in their field and geochemical features such as their relatively high contents of Rb, Th, Nb, Ta, Zr, Hf, Ga, and Y. In the (a) (K_2_O+Na_2_O) (b) Y, (c) Nb, and (d) Zr vs. 10,000 Ga/Al classification diagrams proposed by Whalen et al. (1987) [78], most of the granites are observed to fall into the A-type granite area (Fig 12). The enrichment of Ga relative to Al and the depletion of CaO, Al_2_O_3_, Eu, and Sr may have been caused due to the plentiful plagioclase fractional crystallization that was observed during an earlier stage. High Ga/Al ratios are observed to be a characteristic of several A-type granites, which indicates an extensional setting in Late Jurassic [79, 80].

**Fig 13:**
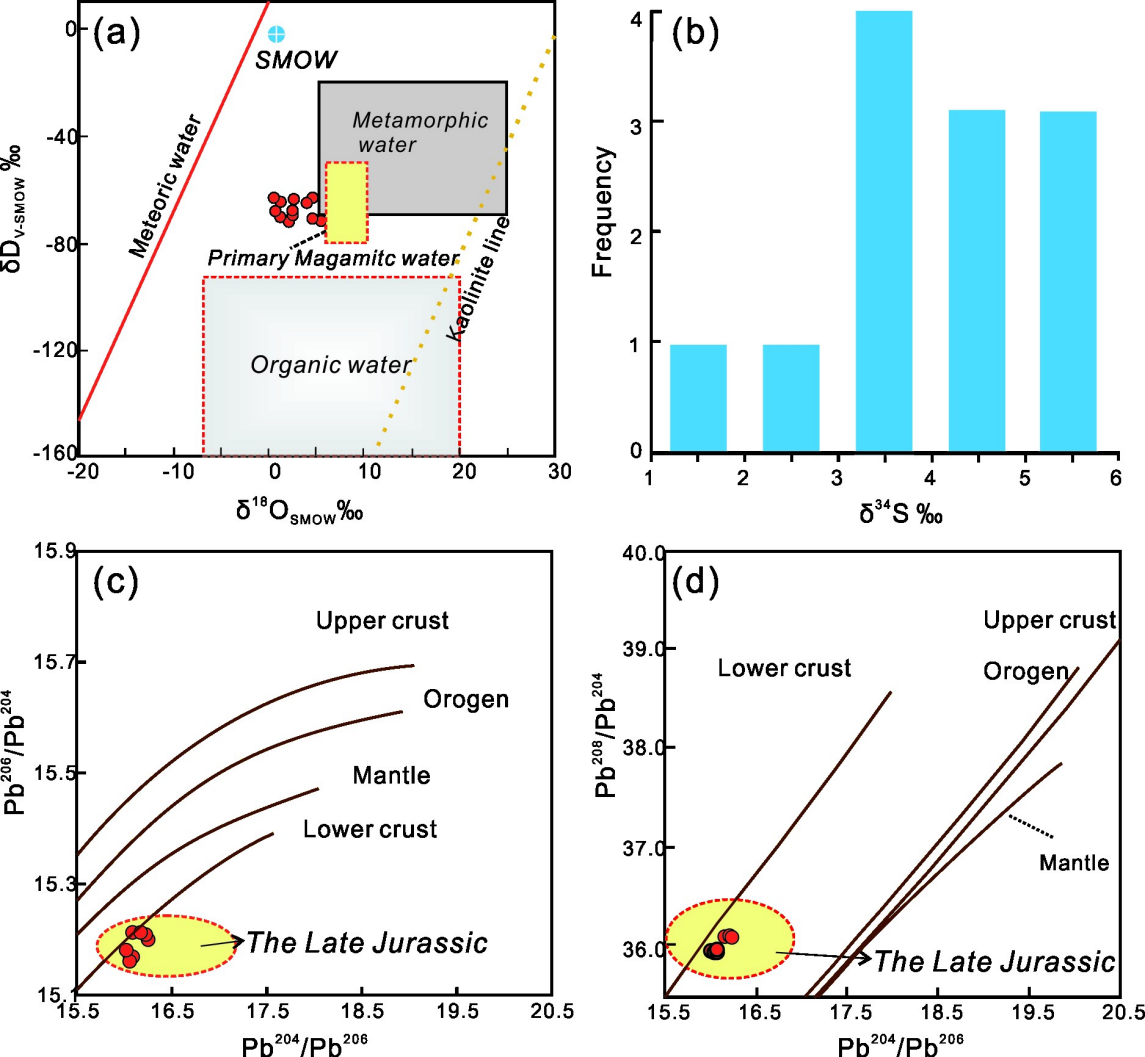
H-O-S-Pb isotope feature of Late Jurassic stage of the NIC. (a)Base map is cited from [81]. Organic water field revised after [82]. (c),(d) Base map from [83].

The S and Pb isotopic compositions of the paragenous sulfide of Late Jurassic granitoids and the H-O isotope compositions of fluid inclusions in the paragenous quartz in NIC (authors’ published H–O–S–Pb isotopic data [28, 29]) indicate that the ore fluids mainly originated from magmatic hydrothermal fluids with the participation of small amounts of meteoric water (Fig 13A). The δ^34^S value of pyrite in the main orebody ranges from 1.5‰ to 5.8‰, having characteristics of crustal-sourced magmatic rock (Fig 13B). The Pb isotope ^206^Pb/^204^Pb composition ranged from 16.02 to 16.25, the ^207^Pb/^204^Pb composition ranged from 15.16 to 15.21, and the ^208^Pb/^204^Pb composition ranged from 35.95 to 36.12. These ranges reflect the typical characteristics of the lower crust lead isotope (Fig 13C and 13D). In the current study, the ore-forming materials were considered to be mainly derived from the Mesozoic-aged magmatic hydrothermal activities. Combining the geochronologic and isotopic features, the mineralization at Huajian was considered to be spatially and temporally related to Late Jurassic (~155 Ma) stage of the NIC and was causatively connected to the emplacement and crystallization of the shallower crustal-sourced part of the intrusive complex.

### Regional tectonic evolution of easten Hebei Province

**Fig 14:**
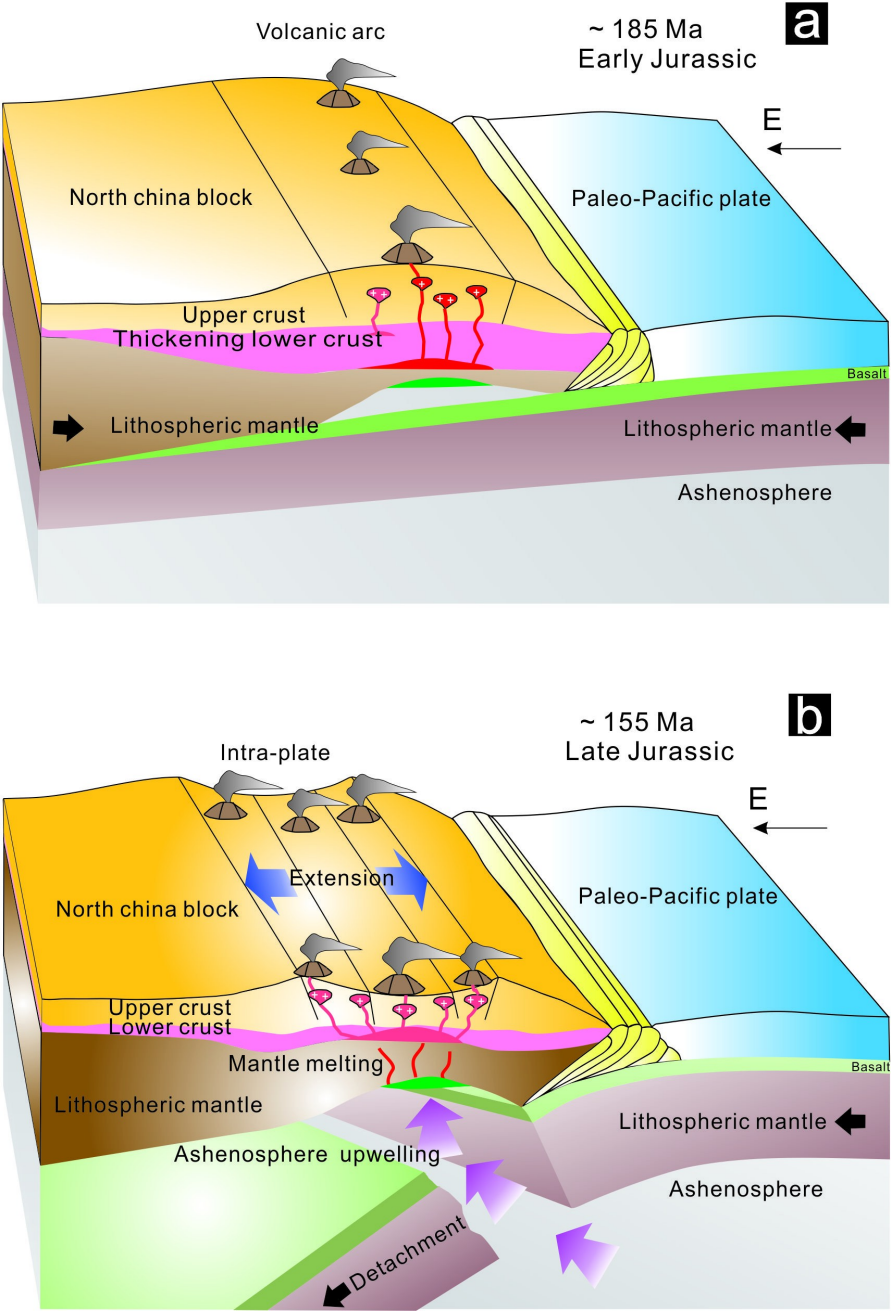
Three-dimensional schematic illustrations of tectonic reconstruction of the northern part of the North China Craton during Early Jurassic (c.185Ma) and Late Jurassic (c.155Ma). (a) Due to the continuous compressing between the NCC and Paleo-Pacific Plate, the Paleo-Pacific Plate subducted far from the coastal area, and gave rise to dehydrated and melted, and generated magma. (b) As the slab dip angle increased, the detachment of the subducted slab and preceding collision between Paleo-Pacific Plate and North China Craton, the upwelling mantle lead to the partial melting of lower crust and generated ore-bearing magma.

By combining geochemistry with the new age data of the NIC, we infer that easten Hebei Province, which includes the Huajian metallogenic district, was generated in a geodynamic environment controlled by the subduction of the Paleo-Pacific Plate beneath the NCC. A two-stage tectonic model is distinguished for the occurrence of the Early to Late Jurassic intrusions in the study area i.e., the thickened crust with compressional setting and the thinned crust with extensional setting (Fig 14). ~185–155 Ma has been constrained as the timing of tectonic regime transition stage on account of the geochronology of NIC. The intense NTT anomalies, high Ba/Th and La/Sm ratios in Early Jurassic igneous rocks are related to the compression by the subduction of the Paleo-Pacific Plate. The negligible NTT anomalies and A-type granites in Late Jurassic igneous rocks are ascribed to the extension by the asthenosphere upwelling.

Prior to ~185 Ma, because of the flat or low angle of the initial subduction of the paleo-Pacific plate, the compressional regime was observed to reoccur in the eastern part of the NCC. The juvenile mafic lower crust was metamorphosed into garnet-bearing gneiss and underwent tectonic thickening. Thickening of the lower crust during the compressional stage, resulting from the partial melting of the lithospheric mantle, generating magma with high Ba:Th and La:Sm and intense NTT anomalies (Fig 14A).

As the velocity and dip angle of the subducted slab increased after ~155 Ma, there were inadequate subducted sediments and fluids to generate magma following the detachment and rollback of the subducted slab. Instead, the upwelling asthenosphere led to the partial melting of the subducted slab, which was then mixed with the ancient lower crust. The hybrid magma ascended to the upper crust, and intensive fractional crystallization or, potentially, upper-crustal assimilation resulted in low Ba:Th and La:Sm, weak NTT anomalies, and A-type granites; the eastern Hebei region was under an extensional tectonic setting with a thinning crust (Fig 14B).

### Effect of tectonic transformation and potential of polymetallic mineralization

The NIC is the pre-eminent example of the connection between hydrothermal-style gold mineralization and shoshonitic magma. It contains all the features that are considered to be optimal for the formation of economic, hydrothermal-style gold deposits, which contain shallowly emplaced high potassium calc-alkaline magmas with petrographic and chemical proof for abundant internal fractionation and depict differentiation that affords the build-up and retention of volatiles and metals in a fraction of the residual melt before volatile exsolution.

The close spatial connection of gold mineralization with the NIC’s A-type granites in the Huajian district suggests a genetic relation. A similar relation is observed in the northern margin of the NCC during the late Mesozoic with A-type granites containing large-scale polymetallic mineralization [84–87]. It is generally assumed that specific tectonic settings are associated with gold–copper–molybdenum mineralization; intra-plate setting related deposits depict similar mineral associations in northeastern NCC [1, 88–90]. The generation of these deposits is assumed to be the result of crustal extension and lithospheric thinning during the Late Jurassic in NE China [19, 32, 36–38, 62, 91, 92].

Many studies have indicated that crustal thickening and shortening with a compressional regime occurred in the NE China during Jurassic time [37, 38, 93, 94]. They are suggested by large-scale contractional deformations and other thrusts along the Solonker–Xar Moron–Changchun–Yanji suture (e.g., [27, 31, 62, 63, 95]), especially the intense widespread Jurassic magmatism–Yanshan orogeny in the northern part of the NCC [96–103]. During Early Cretaceous time, the tectonic regime transformed from compressional to extensional as evidenced by large-scale emergence of the early Cretaceous metamorphic core complexes (MCCs; e.g.,[104–106]), and by a lot of volcanic rocks and extensional basins (e.g.,[107–109]). The initial time of crustal thinning is usually considered to be at 155–140 Ma, interpreted to be related to the subduction of Paleo-Pacific Plate, with peak time at 130–120 Ma ([104, 106]). Moreover, early Cretaceous granites were widespread in northern part of the NCC, indicating extension predominantly occured in early Cretaceous (S3 Table) [23, 105, 110]). These features provide robust proof that Late Jurassic was the transition time of the crustal from thickening to thinning.

Therefore, the newly obtained Zircon U–Pb ages for the NIC indicate that mineralization occurred during Late Jurassic (~155 Ma), which corresponds to an intra-plate extensional environment with a thinned crust in the northern margin of the NCC. As stated previously, the occurrences of the mineralization and the zircon U–Pb ages of the A-type granites indicate that the mineralization was likely be formed by hydrothermal replacement during lithospheric thinning due to hybrid magma ascending to the upper crust, which caused the formation of the A-type granites [67, 68, 78, 80, 92]. Because of the spatial and temporal relations of the Au–Cu–Mo mineralization and the tectonic settings in which it occurs, we consider that the Au–Cu–Mo mineralization of Late Jurassic age along the northern margin of the NCC was likely to be related to lithospheric thinning after the continuing subduction of the paleo-Pacific plate [111–114]. Recent mapping indicates that granites of the Mesozoic age are widespread throughout this region [21, 115–117] S3 Table. Among the mineralization ages along the northern margin of the NCC, the establishment of a Late Jurassic age should stimulate renewed exploration to discover new Au–Cu–Mo resources.

## Conclusions

1. The timing of tectonic regime transition from volcanic arc to intra-plate setting during Late Mesozoic occurred between ~185 to ~155 Ma on account of the geochemistry and the geochronology of NIC and combined isotope data, ~155 Ma magmatism is connected with the mineralization.
2. Geochemical characteristics of two dated granites can be distinguished two types. The first group is characterized by relatively steep REE patterns with slight Eu anomalies low Yb-high Sr. The second group contains flat REE patterns with obvious Eu anomalies, high Yb-low Sr, and weak NTT anomalies.
3. Before ~185 Ma, the volcanic arc convergent setting was probably attributed to subduction of the Paleo-Pacific Plate. After ~155 Ma, due to the increased velocity and dip angle of subducted slab, its main tectonic setting changing to intra-plate extension and lithosphere varying thickening to thinning.

## Supporting information

S1 Table Zircon LA-ICP-MS U-Pb data results of the Niuxinshan intrusive complex. (DOC)

S2 Table Major element (wt%), trace element (ppm), and REE (ppm) composition of the Niuxinshan granitoid. (DOC)

S3 Table Zircon U–Pb ages for Mesozoic (Jurassic–Early Cretaceous) granitoid magmatisms in the Northern North China Craton (NNCC). (DOC)
