# Supplementary material for: Geological significance of new zircon U–Pb geochronology and geochemistry: Niuxinshan intrusive complex, northern North China Craton
Source: PLoS One. 2019 Mar 6;14(3):e0213156. doi: 10.1371/journal.pone.0213156 (PMC6402702; doi:10.1371/journal.pone.0213156)
Supplement: S1 Table — (DOC) [file pone.0213156.s001.doc]

**S1 Table**. Zircon LA-ICP-MS U-Pb data results of the Niuxinshan intrusive complex

| Spot No. | | | U (ppm) | | Th (ppm) | Th/U | ^207^Pb/^206^Pb | | ^207^Pb/^235^U | | ^206^Pb/^238^U | | t(^206^Pb/^238^U) | |
| --- | --- | --- | --- | --- | --- | --- | --- | --- | --- | --- | --- | --- | --- | --- |
|  |  |  |  |  |  |  | Ratio  value | 1σ | Ratio  value | 1σ | Ratio  value | 1σ | Age  (Ma) | 1σ |
| *Sample HNK103-4* | | | |  | |  |  |  |  |  |  |  |  |  |
| **GJ-1** | | | 401 | 7 | | 0.02 | 0.0593 | 0.0009 | 0.7896 | 0.0139 | 0.0966 | 0.0012 | 594 | 7 |
| **HS4-01** | | | 556 | 318 | | 0.57 | 0.0535 | 0.0015 | 0.2153 | 0.0063 | 0.0292 | 0.0004 | 185 | 3 |
| **HS4-02** | | | 675 | 250 | | 0.37 | 0.0533 | 0.0025 | 0.1759 | 0.0079 | 0.024 | 0.0004 | 153 | 2 |
| **HS4-03** | | | 527 | 276 | | 0.52 | 0.0538 | 0.0016 | 0.2143 | 0.0064 | 0.0289 | 0.0004 | 184 | 3 |
| **HS4-04** | | | 791 | 332 | | 0.42 | 0.0513 | 0.0015 | 0.172 | 0.005 | 0.0243 | 0.0003 | 155 | 2 |
| **HS4-05** | | | 702 | 318 | | 0.45 | 0.0524 | 0.0016 | 0.1805 | 0.0055 | 0.025 | 0.0004 | 159 | 2 |
| **HS4-07** | | | 338 | 326 | | 0.96 | 0.0546 | 0.0016 | 0.2198 | 0.0067 | 0.0292 | 0.0004 | 185 | 3 |
| **HS4-09** | | | 744 | 296 | | 0.40 | 0.0508 | 0.0015 | 0.1747 | 0.0054 | 0.0249 | 0.0004 | 159 | 2 |
| **HS4-11** | | | 749 | 307 | | 0.41 | 0.0507 | 0.0016 | 0.1678 | 0.0053 | 0.024 | 0.0004 | 153 | 2 |
| **HS4-12** | | | 895 | 507 | | 0.57 | 0.0615 | 0.002 | 0.2056 | 0.0066 | 0.0242 | 0.0004 | 154 | 2 |
| **HS4-13** | | | 439 | 247 | | 0.56 | 0.0571 | 0.0018 | 0.2298 | 0.0073 | 0.0292 | 0.0004 | 185 | 3 |
| **HS4-15** | | | 1134 | 625 | | 0.55 | 0.0532 | 0.0017 | 0.1761 | 0.0056 | 0.024 | 0.0004 | 153 | 2 |
| **HS4-16** | | | 849 | 317 | | 0.37 | 0.055 | 0.0018 | 0.1857 | 0.006 | 0.0245 | 0.0004 | 156 | 2 |
| **HS4-17** | | | 622 | 344 | | 0.55 | 0.0517 | 0.0017 | 0.2067 | 0.0067 | 0.029 | 0.0004 | 184 | 3 |
| **HS4-18** | | | 581 | 379 | | 0.65 | 0.0519 | 0.0017 | 0.2102 | 0.0068 | 0.0294 | 0.0004 | 187 | 3 |
| **HS4-19** | | | 440 | 296 | | 0.67 | 0.057 | 0.0019 | 0.2231 | 0.0075 | 0.0284 | 0.0004 | 180 | 3 |
| **HS4-20** | | | 948 | 585 | | 0.62 | 0.0565 | 0.0019 | 0.1881 | 0.0063 | 0.0241 | 0.0004 | 154 | 2 |
| **HS4-21** | | | 484 | 291 | | 0.60 | 0.0567 | 0.0019 | 0.229 | 0.0078 | 0.0293 | 0.0004 | 186 | 3 |
| **HS4-22** | | | 828 | 413 | | 0.50 | 0.056 | 0.0019 | 0.2293 | 0.0077 | 0.0297 | 0.0004 | 189 | 3 |
| **HS4-23** | | | 423 | 186 | | 0.44 | 0.056 | 0.0019 | 0.2288 | 0.0078 | 0.0296 | 0.0004 | 188 | 3 |
| **HS4-24** | | | 830 | 355 | | 0.43 | 0.0623 | 0.0021 | 0.2089 | 0.0072 | 0.0243 | 0.0004 | 155 | 2 |
| **HS4-25** | | | 637 | 331 | | 0.52 | 0.0537 | 0.0019 | 0.1778 | 0.0062 | 0.024 | 0.0004 | 153 | 2 |
| **HS4-26** | | | 1015 | 558 | | 0.55 | 0.0567 | 0.0019 | 0.2302 | 0.0079 | 0.0295 | 0.0004 | 187 | 3 |
| **HS4-27** | | | 630 | 408 | | 0.65 | 0.0554 | 0.0019 | 0.2227 | 0.0078 | 0.0291 | 0.0004 | 185 | 3 |
| **HS4-28** | | | 1127 | 578 | | 0.51 | 0.0507 | 0.0017 | 0.2042 | 0.0071 | 0.0292 | 0.0004 | 185 | 3 |
| **GJ-1** | | | 406 | 9 | | 0.02 | 0.0581 | 0.0008 | 0.7762 | 0.0133 | 0.0971 | 0.0012 | 598 | 7 |
| *Sample HNK103-7* | | | |  | |  |  |  |  |  |  |  |  |  |
| **GJ-1** | | | 1351 | 38 | | 0.03 | 0.0598 | 0.0013 | 0.8046 | 0.0197 | 0.0976 | 0.0013 | 600 | 8 |
| **HS7-01** | | | 724 | 288 | | 0.40 | 0.00762 | 0.0001 | 0.1866 | 0.0039 | 0.0245 | 0.00031 | 156 | 8 |
| **HS7-02** | | | 175 | 164 | | 0.94 | 0.13096 | 0.0015 | 11.1296 | 0.2508 | 0.4785 | 0.00603 | 2521 | 2 |
| **HS7-03** | | | 900 | 399 | | 0.44 | 0.00753 | 0.0001 | 0.1863 | 0.004 | 0.0243 | 0.0003 | 155 | 26 |
| **HS7-04** | | | 29 | 26 | | 0.90 | 0.10621 | 0.0012 | 8.51 | 0.1997 | 0.372 | 0.0047 | 2039 | 2 |
| **HS7-05** | | | 905 | 403 | | 0.45 | 0.00763 | 0.0001 | 0.1833 | 0.0039 | 0.0248 | 0.00031 | 158 | 22 |
| **HS7-06** | | | 820 | 451 | | 0.55 | 0.00686 | 0.0001 | 0.1968 | 0.0043 | 0.0252 | 0.00032 | 160 | 2 |
| **HS7-07** | | | 404 | 252 | | 0.62 | 0.00757 | 0.0001 | 0.2106 | 0.0047 | 0.0244 | 0.00031 | 155 | 2 |
| **HS7-08** | | | 712 | 382 | | 0.54 | 0.00757 | 0.0001 | 0.1826 | 0.0041 | 0.0249 | 0.00031 | 158 | 2 |
| **HS7-09** | | | 859 | 369 | | 0.43 | 0.00734 | 0.0001 | 0.183 | 0.004 | 0.0247 | 0.00031 | 157 | 2 |
| **HS7-10** | | | 791 | 310 | | 0.39 | 0.00777 | 0.0001 | 0.1733 | 0.0057 | 0.0247 | 0.00031 | 157 | 2 |
| **HS7-12** | | | 291 | 198 | | 0.68 | 0.00764 | 0.0001 | 0.3012 | 0.0069 | 0.0295 | 0.00037 | 187 | 2 |
| **HS7-13** | | | 712 | 463 | | 0.65 | 0.00605 | 0.0001 | 0.2229 | 0.005 | 0.0295 | 0.00037 | 188 | 2 |
| **HS7-14** | | | 528 | 310 | | 0.59 | 0.00664 | 0.0001 | 0.2285 | 0.0052 | 0.0287 | 0.00036 | 182 | 2 |
| **HS7-15** | | | 556 | 321 | | 0.58 | 0.00646 | 0.0001 | 0.228 | 0.0052 | 0.0287 | 0.00036 | 182 | 2 |
| **HS7-16** | | | 732 | 303 | | 0.41 | 0.00737 | 0.0001 | 0.175 | 0.004 | 0.0236 | 0.0003 | 150 | 2 |
| **HS7-17** | | | 495 | 254 | | 0.51 | 0.00753 | 0.0001 | 0.1841 | 0.0043 | 0.0244 | 0.00031 | 156 | 2 |
| **HS7-18** | | | 763 | 378 | | 0.49 | 0.00764 | 0.0001 | 0.1793 | 0.0043 | 0.0237 | 0.0003 | 151 | 2 |
| **HS7-19** | | | 887 | 317 | | 0.36 | 0.00767 | 0.0001 | 0.1805 | 0.0042 | 0.024 | 0.0003 | 153 | 2 |
| **HS7-20** | | | 111 | 120 | | 1.08 | 0.00644 | 0.0001 | 0.2519 | 0.007 | 0.0297 | 0.00039 | 188 | 2 |
| **HS7-21** | | | 775 | 253 | | 0.33 | 0.00752 | 0.0001 | 0.1743 | 0.0042 | 0.0237 | 0.0003 | 151 | 2 |
| **HS7-22** | | | 611 | 269 | | 0.44 | 0.0074 | 0.0001 | 0.2253 | 0.0054 | 0.0295 | 0.00037 | 187 | 2 |
| **HS7-23** | | | 907 | 405 | | 0.45 | 0.00762 | 0.0001 | 0.1806 | 0.0043 | 0.0237 | 0.0003 | 151 | 2 |
| **HS7-24** | | | 794 | 313 | | 0.39 | 0.00732 | 0.0001 | 0.1821 | 0.0045 | 0.0242 | 0.00031 | 154 | 2 |
| **HS7-25** | | | 472 | 330 | | 0.70 | 0.00612 | 0.0001 | 0.2549 | 0.0063 | 0.0298 | 0.00038 | 189 | 2 |
| **HS7-26** | | | 425 | 272 | | 0.64 | 0.00729 | 0.0001 | 0.2736 | 0.0068 | 0.0293 | 0.00038 | 186 | 2 |
| **HS7-27** | | | 559 | 398 | | 0.71 | 0.01056 | 0.0001 | 0.2384 | 0.0059 | 0.0293 | 0.00037 | 186 | 2 |
| **HS7-28** | | | 1389 | 2395 | | 1.72 | 0.00682 | 0.0001 | 0.1733 | 0.0042 | 0.0241 | 0.00031 | 154 | 2 |
| **GJ-1** | | | 1348 | 64 | | 0.05 | 0.0608 | 0.0010 | 0.8224 | 0.0163 | 0.0982 | 0.0012 | 604 | 2 |
| *Sample HNK103-16* | | |  |  | |  |  |  |  |  |  |  |  |  |
| **GJ-1** | 440 | | | 11 | | 0.03 | 0.0601 | 0.0008 | 0.8146 | 0.0115 | 0.0983 | 0.0010 | 604 | 7 |
| **HS16-01** | | 705 | | 1394 | | 1.98 | 0.0512 | 0.0017 | 0.2075 | 0.0069 | 0.0294 | 0.0004 | 187 | 6 |
| **HS16-02** | | 380 | | 427 | | 1.12 | 0.0565 | 0.0026 | 0.2275 | 0.0103 | 0.0292 | 0.0005 | 185 | 3 |
| **HS16-03** | | 341 | | 319 | | 0.94 | 0.0512 | 0.0028 | 0.2058 | 0.0111 | 0.0292 | 0.0005 | 185 | 3 |
| **HS16-04** | | 279 | | 294 | | 1.05 | 0.0509 | 0.0045 | 0.2035 | 0.0176 | 0.0290 | 0.0005 | 184 | 3 |
| **HS16-05** | | 835 | | 1853 | | 2.22 | 0.0547 | 0.0017 | 0.2216 | 0.0068 | 0.0294 | 0.0004 | 187 | 3 |
| **HS16-06** | | 446 | | 614 | | 1.38 | 0.0517 | 0.0022 | 0.2103 | 0.0090 | 0.0295 | 0.0004 | 188 | 3 |
| **HS16-07** | | 714 | | 1306 | | 1.83 | 0.0513 | 0.0018 | 0.2069 | 0.0072 | 0.0292 | 0.0004 | 186 | 3 |
| **HS16-08** | | 872 | | 998 | | 1.15 | 0.0509 | 0.0016 | 0.1308 | 0.0056 | 0.0258 | 0.0004 | 164 | 3 |
| **HS16-09** | | 876 | | 1554 | | 1.77 | 0.0517 | 0.0017 | 0.2087 | 0.0069 | 0.0293 | 0.0004 | 186 | 2 |
| **HS16-10** | | 598 | | 963 | | 1.61 | 0.0520 | 0.0019 | 0.2104 | 0.0077 | 0.0293 | 0.0004 | 186 | 3 |
| **HS16-11** | | 645 | | 1460 | | 2.26 | 0.0507 | 0.0020 | 0.2061 | 0.0082 | 0.0295 | 0.0005 | 187 | 3 |
| **HS16-12** | | 411 | | 501 | | 1.22 | 0.0530 | 0.0024 | 0.2113 | 0.0093 | 0.0289 | 0.0005 | 184 | 3 |
| **HS16-13** | | 1407 | | 4776 | | 3.39 | 0.0522 | 0.0015 | 0.2103 | 0.0060 | 0.0292 | 0.0004 | 185 | 3 |
| **HS16-14** | | 1109 | | 3588 | | 3.24 | 0.0518 | 0.0015 | 0.2100 | 0.0062 | 0.0294 | 0.0004 | 187 | 3 |
| **HS16-15** | | 818 | | 1747 | | 2.14 | 0.0517 | 0.0017 | 0.2067 | 0.0069 | 0.0290 | 0.0004 | 184 | 3 |
| **HS16-16** | | 322 | | 429 | | 1.33 | 0.0545 | 0.0070 | 0.2162 | 0.0273 | 0.0288 | 0.0007 | 183 | 3 |
| **HS16-17** | | 446 | | 631 | | 1.42 | 0.0524 | 0.0017 | 0.2084 | 0.0069 | 0.0288 | 0.0004 | 183 | 4 |
| **HS16-18** | | 531 | | 600 | | 1.13 | 0.0483 | 0.0047 | 0.1321 | 0.0173 | 0.0274 | 0.0005 | 174 | 3 |
| **HS16-19** | | 875 | | 1559 | | 1.78 | 0.0546 | 0.0019 | 0.2170 | 0.0074 | 0.0288 | 0.0004 | 183 | 3 |
| **HS16-20** | | 655 | | 1378 | | 2.10 | 0.0504 | 0.0016 | 0.2028 | 0.0064 | 0.0292 | 0.0004 | 185 | 3 |
| **HS16-21** | | 783 | | 1400 | | 1.79 | 0.0510 | 0.0016 | 0.2022 | 0.0062 | 0.0288 | 0.0004 | 183 | 3 |
| **HS16-22** | | 300 | | 360 | | 1.20 | 0.0531 | 0.0022 | 0.2102 | 0.0087 | 0.0287 | 0.0005 | 183 | 3 |
| **HS16-23** | | 250 | | 255 | | 1.02 | 0.0553 | 0.0024 | 0.2163 | 0.0094 | 0.0284 | 0.0005 | 180 | 3 |
| **HS16-24** | | 886 | | 2438 | | 2.75 | 0.0518 | 0.0016 | 0.2034 | 0.0063 | 0.0285 | 0.0004 | 181 | 3 |
| **HS16-25** | | 495 | | 866 | | 1.75 | 0.0533 | 0.0019 | 0.2143 | 0.0078 | 0.0291 | 0.0004 | 185 | 3 |
| **HS16-26** | | 1092 | | 2278 | | 2.09 | 0.0519 | 0.0020 | 0.2069 | 0.0080 | 0.0289 | 0.0004 | 184 | 3 |
| **HS16-27** | | 351 | | 425 | | 1.21 | 0.0494 | 0.0019 | 0.1368 | 0.0173 | 0.0274 | 0.0004 | 174 | 3 |
| **HS16-28** | | 801 | | 1613 | | 2.01 | 0.0525 | 0.0017 | 0.2089 | 0.0066 | 0.0289 | 0.0004 | 183 | 3 |
| **HS16-29** | | 633 | | 1165 | | 1.84 | 0.0513 | 0.0017 | 0.2068 | 0.0068 | 0.0292 | 0.0004 | 186 | 3 |
| **GJ-1** | | 441 | | 12 | | 0.03 | 0.0602 | 0.0008 | 0.8040 | 0.0111 | 0.0969 | 0.0010 | 596 | 3 |
