# Supplementary material for: Geological significance of new zircon U–Pb geochronology and geochemistry: Niuxinshan intrusive complex, northern North China Craton
Source: PLoS One. 2019 Mar 6;14(3):e0213156. doi: 10.1371/journal.pone.0213156 (PMC6402702; doi:10.1371/journal.pone.0213156)
Supplement: S2 Table — (DOC) [file pone.0213156.s002.doc]

**S2 Table**. Major element (wt%), trace element (ppm), and REE (ppm) composition of the Niuxinshan granitoid

| Sample | HNK103-H12 | HNK103-H8 | HNK103-H14 | HNK103-H21 | HNK103-H19 | HNK103-H13 | HNK103-H17 | HNK103-H18 | HNK103-H23 | HNK103-H24’ | HNK103-H24 | HNK103-H25 | HNK105-H4 |
| --- | --- | --- | --- | --- | --- | --- | --- | --- | --- | --- | --- | --- | --- |
| SiO_2_ | 73.98 | 73.68 | 73.83 | 74.25 | 70.65 | 72.58 | 73.93 | 74.12 | 74.67 | 73.68 | 76.98 | 52.16 | 62.75 |
| TiO_2_ | 0.07 | 0.06 | 0.06 | 0.05 | 0.06 | 0.07 | 0.06 | 0.07 | 0.07 | 0.06 | 0.08 | 0.07 | 0.08 |
| Al_2_O_3_ | 13.33 | 13.23 | 13.33 | 13.01 | 14.55 | 14.03 | 12.89 | 13.42 | 12.95 | 13.23 | 11.73 | 13.31 | 16.07 |
| Fe_2_O_3_^T^ | 0.95 | 0.77 | 0.78 | 0.84 | 0.76 | 1.57 | 1.01 | 0.72 | 0.64 | 0.77 | 0.46 | 7.70 | 4.21 |
| MnO | 0.26 | 0.26 | 0.26 | 0.22 | 0.30 | 0.48 | 0.22 | 0.23 | 0.23 | 0.26 | 0.03 | 0.13 | 0.32 |
| MgO | 0.15 | 0.09 | 0.10 | 0.15 | 0.20 | 0.47 | 0.27 | 0.11 | 0.10 | 0.09 | 0.13 | 4.06 | 2.24 |
| CaO | 0.96 | 1.31 | 1.24 | 0.85 | 2.67 | 2.23 | 1.55 | 1.18 | 1.28 | 1.31 | 0.58 | 4.16 | 1.71 |
| Na_2_O | 1.95 | 2.47 | 2.52 | 3.71 | 0.40 | 0.23 | 2.15 | 2.07 | 3.51 | 2.47 | 2.83 | 2.83 | 1.01 |
| K_2_O | 5.97 | 5.67 | 5.66 | 5.01 | 7.46 | 5.01 | 5.79 | 6.76 | 5.06 | 5.67 | 5.57 | 3.15 | 6.29 |
| P_2_O_5_ | 0.02 | 0.04 | 0.01 | 0.04 | 0.01 | 0.01 | 0.01 | 0.02 | 0.02 | 0.04 | 0.03 | 0.17 | 0.16 |
| LOI | 1.54 | 1.83 | 1.52 | 1.09 | 2.53 | 2.38 | 1.42 | 1.19 | 1.40 | 1.83 | 0.71 | 11.21 | 4.19 |
| Total | 99.18 | 99.41 | 99.31 | 99.22 | 99.59 | 99.06 | 99.3 | 99.89 | 99.93 | 99.41 | 99.12 | 98.95 | 99.03 |
| Ga | 23.24 | 22.20 | 22.38 | 23.26 | 20.10 | 24.26 | 22.90 | 21.68 | 22.58 | 15.65 | 15.37 | 17.08 | 19.48 |
| Rb | 324.20 | 277.80 | 291.00 | 292.60 | 783.60 | 218.20 | 342.00 | 361.20 | 337.00 | 130.34 | 129.40 | 122.6 | 140.2 |
| Sr | 53.06 | 36.62 | 38.12 | 41.34 | 65.26 | 27.00 | 58.00 | 64.02 | 60.32 | 88.02 | 100.34 | 168.8 | 105.12 |
| Y | 36.84 | 35.28 | 35.28 | 40.88 | 38.76 | 40.86 | 36.14 | 32.84 | 41.88 | 12.62 | 12.74 | 7.64 | 7.71 |
| Zr | 96.52 | 84.22 | 80.02 | 83.90 | 88.90 | 103.30 | 90.20 | 88.88 | 105.54 | 102.66 | 114.58 | 106.36 | 103.51 |
| Nb | 110.46 | 102.22 | 98.08 | 103.64 | 118.82 | 118.00 | 113.58 | 106.12 | 143.62 | 13.18 | 19.04 | 10.79 | 8.72 |
| Ba | 224.00 | 189.88 | 202.20 | 195.14 | 497.00 | 154.98 | 248.20 | 300.00 | 162.70 | 118.36 | 141.22 | 355.20 | 296.40 |
| La | 8.06 | 4.48 | 6.46 | 5.89 | 6.86 | 6.96 | 6.81 | 8.23 | 8.60 | 15.89 | 15.42 | 18.65 | 29.40 |
| Ce | 19.95 | 11.30 | 15.97 | 14.80 | 16.98 | 17.27 | 17.01 | 20.38 | 21.66 | 28.92 | 28.16 | 37.16 | 58.26 |
| Pr | 2.73 | 1.60 | 2.19 | 2.05 | 2.35 | 2.38 | 2.36 | 2.80 | 3.01 | 2.82 | 2.74 | 4.33 | 6.84 |
| Nd | 11.68 | 7.33 | 9.58 | 9.10 | 10.18 | 10.34 | 10.34 | 11.98 | 12.80 | 8.66 | 8.39 | 16.57 | 26.36 |
| Sm | 4.16 | 3.43 | 3.70 | 3.67 | 3.73 | 4.01 | 3.99 | 4.14 | 4.70 | 3.2 | 3.53 | 2.67 | 4.25 |
| Eu | 0.26 | 0.24 | 0.25 | 0.23 | 0.29 | 0.26 | 0.26 | 0.28 | 0.27 | 0.92 | 0.9 | 0.90 | 0.88 |
| Gd | 4.85 | 4.66 | 4.68 | 4.77 | 4.70 | 5.20 | 4.85 | 4.62 | 5.40 | 2.6 | 3.1 | 2.16 | 3.06 |
| Tb | 0.87 | 0.85 | 0.88 | 0.87 | 0.82 | 0.97 | 0.86 | 0.78 | 0.94 | 0.28 | 0.26 | 0.25 | 0.32 |
| Dy | 5.45 | 5.33 | 5.49 | 5.63 | 5.23 | 6.17 | 5.42 | 4.84 | 5.76 | 1.86 | 1.77 | 1.36 | 1.59 |
| Ho | 0.98 | 0.96 | 0.97 | 1.02 | 0.97 | 1.10 | 0.96 | 0.87 | 1.03 | 0.37 | 0.36 | 0.24 | 0.27 |
| Er | 2.79 | 2.77 | 2.82 | 2.97 | 2.89 | 3.24 | 2.81 | 2.57 | 3.01 | 1.18 | 1.18 | 0.72 | 0.74 |
| Tm | 0.41 | 0.41 | 0.41 | 0.44 | 0.43 | 0.48 | 0.42 | 0.39 | 0.46 | 0.18 | 0.18 | 0.09 | 0.09 |
| Yb | 2.92 | 2.93 | 2.97 | 3.20 | 3.18 | 3.40 | 3.02 | 2.80 | 3.37 | 1.34 | 1.37 | 0.66 | 0.64 |
| Lu | 0.41 | 0.41 | 0.41 | 0.45 | 0.46 | 0.48 | 0.43 | 0.40 | 0.49 | 0.19 | 0.20 | 0.09 | 0.09 |
| Hf | 4.76 | 4.02 | 3.89 | 4.52 | 4.40 | 4.85 | 4.44 | 4.24 | 5.28 | 3.49 | 3.75 | 3.80 | 3.22 |
| Ta | 17.69 | 17.33 | 15.21 | 18.61 | 21.98 | 19.80 | 20.32 | 17.73 | 25.84 | 0.41 | 0.55 | 0.43 | 0.39 |
| Pb | 124.86 | 51.60 | 47.44 | 44.72 | 1956.40 | 17.10 | 52.82 | 47.30 | 24.92 | 29.40 | 17.68 | 10.78 | 27.90 |
| Th | 34.20 | 31.70 | 32.58 | 30.52 | 33.04 | 35.28 | 32.00 | 33.88 | 34.72 | 9.54 | 8.61 | 7.28 | 6.2 |
| U | 18.78 | 12.73 | 16.28 | 17.41 | 15.07 | 34.82 | 15.28 | 16.04 | 17.63 | 2.59 | 2.19 | 5.71 | 0.49 |
