# Supplementary material for: Geological significance of new zircon U–Pb geochronology and geochemistry: Niuxinshan intrusive complex, northern North China Craton
Source: PLoS One. 2019 Mar 6;14(3):e0213156. doi: 10.1371/journal.pone.0213156 (PMC6402702; doi:10.1371/journal.pone.0213156)
Supplement: S3 Table — (DOC) [file pone.0213156.s003.doc]

**S3 Table. Zircon U–Pb ages for Mesozoic (Jurassic–Early Cretaceous) granitoid magmatisms in the Northern North China Craton (NNCC).**

| **Sample** | **Tectonic location** | **Pluton name** | **Lithology** | **Age (Ma)** | **Method** | **References** |
| --- | --- | --- | --- | --- | --- | --- |
| XJY | NNCC | Xiaojiayingzi | Syengranite | 170 ± 1 | SHRIMP |  |
| Heixiongshan | NNCC | Heixiongshan | Granite | 124 ± 1.1 | SHRIMP |  |
| Humen | NNCC | Humen | Quartz monzodiorite | 124 ± 1.8 | SHRIMP |  |
| Heishanzhai | NNCC | Heishanzhai | Granodiorite | 125 ± 1.5 | SHRIMP |  |
| Baicha | NNCC | Baicha | Granite | 127 ± 0.7 | SHRIMP |  |
| Xuejiashiliang | NNCC | Xuejiashiliang | Monzogranite | 130 ± 1.7 | SHRIMP |  |
| Hanjiachuan | NNCC | Hanjiachuan | Monzogranite | 138 ± 1.2 | SHRIMP |  |
| Duijiuyu | NNCC | Duijiuyu | Monzogranite | 138 ± 3.6 | SHRIMP |  |
| Yunmengshan | NNCC | Yunmengshan | Two-mica granite | 145 ± 2.7 | SHRIMP |  |
| Changyuan | NNCC | Changyuan | Two-mica granite | 153 ± 3.2 | SHRIMP |  |
| Shicheng | NNCC | Shicheng | Granite Dyke | 156 ± 1.5 | SHRIMP |  |
| Siganding | NNCC | Siganding | Two-mica Granite | 159 ± 1.9 | SHRIMP |  |
| Dashipo | NNCC | Dashipo | Granodiorite enclave | 197 ± 1.9 | SHRIMP |  |
| BHG4 | NNCC | Duimiangou | Monzogranite | 128 ± 1 | LA-ICP-MS |  |
| XL03 | NNCC | Xinglonggou | Quartz monzodiorite | 144 ± 9 | SHRIMP |  |
| DQ08-100 | NNCC | Shenshuiling | Monzogranite | 140 ± 1 | LA-ICP-MS |  |
| DQ08-110 | NNCC | Shenshuiling | Bi-granite | 148 ± 1 | LA-ICP-MS |  |
| DQ08-38 | NNCC | Kuisucun | Bi-monzogranite | 142 ± 1 | LA-ICP-MS |  |
| DQ08-65-1 | NNCC | Daqingshan | Granodiorite | 142 ± 1 | SHRIMP |  |
| DQ08-68-7 | NNCC | Daqingshan | Alkali feldspar | 132 ± 2 | LA-ICP-MS |  |
| DQ08-70 | NNCC | Kuisucun | Monzogranite | 114 ± 1 | LA-ICP-MS |  |
| Hu09-55 | NNCC | Shenshuiling | Quartzdiorite | 138 ± 1 | LA-ICP-MS |  |
| JN0743 | NNCC | Shangshuiquan | Monzogranite | 143 ± 1 | LA-ICP-MS |  |
| LJ023 | NNCC | Jiuliancheng | Monzogranite | 157 ± 6 | SHRIMP |  |
| LJ037 | NNCC | Gaoliduntai | Syenogranite | 156 ± 5 | SHRIMP |  |
| SHSHQ-1 | NNCC | Shangshuiquan | Monzogranite | 142 ± 1 | SHRIMP |  |
| 07FS02 | NNCC | Fangshan | Monzogranite | 132 ± 2 | LA-ICP-MS |  |
| 07FS09 | NNCC | Fangshan | Bi-amphibole plagiogneiss | 130 ± 1 | LA-ICP-MS |  |
| 07FS11 | NNCC | Fangshan | Bi-granite | 134 ± 2 | LA-ICP-MS |  |
| Ln09630-10 | NNCC | Liaonan | Quartz-monzonite porphyry | 115 ± 1 | LA-ICP-MS |  |
| Ln09630-11.1 | NNCC | Wanfu | Syenogranite | 170 ± 1 | LA-ICP-MS |  |
| Ln805012b | NNCC | Liaonan | Quartz porphyry | 129 ± 2 | LA-ICP-MS |  |
| 05FW064 | NNCC | Jingpeng | Syenogranite | 141 ± 1 | LA-ICP-MS |  |
| F04-033 | NNCC | Xiangshan | Monzogranite | 117 ± 1 | LA-ICP-MS |  |
| F04-067 | NNCC | Houhushan | Quartz-monzonite porphyry | 120 ± 1 | LA-ICP-MS |  |
| F04-073 | NNCC | Houhushan | Monzogranite | 118 ± 1 | LA-ICP-MS |  |
| F04-106 | NNCC | Qiancengbei | Granodiorite porphyry | 129 ± 1 | LA-ICP-MS |  |
| F04-111 | NNCC | Wulingshan | Granite porphyry | 129 ± 1 | LA-ICP-MS |  |
| F04-113 | NNCC | Wulingshan | Granite porphyry | 130 ± 1 | LA-ICP-MS |  |
| F04-114 | NNCC | Wulingshan | Granite porphyry | 130 ± 1 | LA-ICP-MS |  |
| FX18 | NNCC | Gangjia | Granodiorite | 153 ± 5 | SHRIMP |  |
| HP9 | NNCC | Hengshan | Granodiorite | 167 ± 6 | SHRIMP |  |
| 1021 | NNCC | Qianzhangzi | Bi-Granite | 164 ± 4 | SHRIMP |  |
| 07D010-1 | NNCC | Zhoujiawopu | Pegmatite | 159 ± 2 | LA-ICP-MS |  |
| 07D044-1 | NNCC | Gangzi | Pegmatite | 138 ± 2 | LA-ICP-MS |  |
| 07D046-1 | NNCC | Jianchang | Pegmatite | 171 ± 3 | LA-ICP-MS |  |
| 08008-1 | NNCC | Chengde | Pegmatite | 159 ± 2 | LA-ICP-MS |  |
| 09018-1 | NNCC | Nianziyu | Pegmatite | 165 ± 2 | LA-ICP-MS |  |
| 09043-1 | NNCC | Liuguanyingzi | Pegmatite | 124 ± 2 | LA-ICP-MS |  |
| 09061-1 | NNCC | Chengjiawopu | Peralkaline granite | 164 ± 1 | LA-ICP-MS |  |
| 10018-1 | NNCC | Shouwangfen | Sheared amphibole–Bi-quartz diorite | 133 ± 1 | LA-ICP-MS |  |
| 10036-1 | NNCC | Chengde | Hornblende subalkali quartz diorite | 161 ± 2 | LA-ICP-MS |  |
| 10096-1 | NNCC | Yunwushan | Hornblende subalkali quartz diorite | 138 ± 1 | LA-ICP-MS |  |
| HPQ020817 | NNCC | Qianzhangzi | Peralkaline granite | 166 ± 2 | SHRIMP |  |
| LLX020811 | NNCC | Xingzhangzi | Two-Mica pegmatite | 165 ± 2 | SHRIMP |  |
| SGD-1 | NNCC | Siganding | Bt-amph granite | 160 ± 5 | SHRIMP |  |
| Fangshan | NNCC | Fangshan | Granosyenite | 131 ± 1 | SHRIMP |  |
| ZKD | NNCC | Fangshan | Leucocratic granites | 131 ± 1 | SHRIMP |  |
| ZB-1 | NNCC | Banlashan | Granite | 132 ± 1 | SHRIMP |  |
| Lanjiagou | NNCC | Lanjiagou | Mylonitic granite | 189 ± 1 | SHRIMP |  |
| DB101 | NNCC | Huanghuacheng | Bi-monzogranite | 133 ± 1 | LA-ICP-MS |  |
| DB104 | NNCC | Fenshuiling | Bi-monzogranite | 129 ± 1 | LA-ICP-MS |  |
| DB107-2 | NNCC | Tieluzi | Bi-monzogranite | 137 ± 1 | LA-ICP-MS |  |
| DB101 | NNCC | Huanghuacheng | Granite porphyry | 133 ± 1 | LA-ICP-MS |  |
| DB104 | NNCC | Fenshuiling | Bi-plagiogranite | 129 ± 1 | LA-ICP-MS |  |
| DB107-2 | NNCC | Tieluzi | Monzogranite | 137 ± 1 | LA-ICP-MS |  |
| DH-23 | NNCC | Daolanghuduge | Syenogranite | 139 ± 2 | SHRIMP |  |
| SBZ09-17 | NNCC | Sibozi-Liubozi | Granite porphyry | 190 ± 1 | LA-ICP-MS |  |
| SBZ09-33 | NNCC | Sibozi-Liubozi | Monzogranite | 160 ± 1 | LA-ICP-MS |  |
| SBZ09-51 | NNCC | Sibozi-Liubozi | Monzogranite | 177 ± 1 | LA-ICP-MS |  |
| SBZ09-56 | NNCC | Sibozi-Liubozi | Granite porphyry | 196 ± 1 | LA-ICP-MS |  |
| WY-48 | NNCC | Yunmengshan | Gneissic granite | 144 ± 4 | SHRIMP |  |
| No. 1 | NNCC | Hongdunliang | Granite | 144 ± 2 | SHRIMP |  |
| No. 4 | NNCC | Donggoulou | Granite | 138 ± 2 | SHRIMP |  |
| 1 | NNCC | Hongdunliang | Bi-monzogranite | 144 ± 2 | SHRIMP |  |
| 4 | NNCC | Donggoulou | Bi-monzogranite | 138 ± 2 | SHRIMP |  |
| QSHK-2 | NNCC | Qingshankou | Granite | 199 ± 2 | SHRIMP |  |
| NXSH-2 | NNCC | Niuxinshan | Granite | 172 ± 2 | SHRIMP |  |
| YEY-3 | NNCC | Yuerya | Granite | 175 ± 1 | SHRIMP |  |
| YEY-8 | NNCC | Yuerya | Granite | 174 ± 3 | SHRIMP |  |
| PSHL-1 | NNCC | Paishanlou | Dioritic porphyrite | 126 ± 2 | SHRIMP |  |
| PSHL-10 | NNCC | Paishanlou | Dioritic porphyrite | 125 ± 1 | SHRIMP |  |
| PSHL-2 | NNCC | Paishanlou | Granite porphyry | 124 ± 1 | SHRIMP |  |
| PSHL-4 | NNCC | Paishanlou | Bi-granite | 124 ± 1 | SHRIMP |  |
| NXSH-2 | NNCC | Niuxinshan | Monzogranite | 172 ± 2 | SHRIMP |  |
| PSHL-1 | NNCC | Paishanlou | Dioritic porphyrite dyke | 126 ± 2 | SHRIMP |  |
| PSHL-10 | NNCC | Paishanlou | Dioritic porphyrite | 125 ± 1 | SHRIMP |  |
| PSHL-2 | NNCC | Paishanlou | Granite porphyry dyke | 124 ± 1 | SHRIMP |  |
| QSHAK-2 | NNCC | Qingshankou | Bi-granite | 199 ± 2 | SHRIMP |  |
| YEY-3 | NNCC | Yuerya | Granite porphyry | 175 ± 1 | SHRIMP |  |
| HDMG-19 | NNCC | Hadamengou | Potassic- plteration Rock | 132 ± 2 | SHRIMP |  |
| EDG-3 | NNCC | Xiduimiangou | Granodiorite-porphyry dyke | 126 ± 1 | SHRIMP |  |
| EDG-7 | NNCC | Loushang | Pyroxene quartzdiorite | 161 ± 1 | SHRIMP |  |
| WB08-N3 | NNCC | Narenwula | Alkali-feldspar Granite | 145 ± 1 | LA-ICP-MS |  |
| WB08-N8 | NNCC | Baiqi | Granite porphyry | 135 ± 1 | LA-ICP-MS |  |
| ZK150-19 | NNCC | Luoguhe | Monzogranite | 131 ± 2 | LA-ICP-MS |  |
| CF06-042 | NNCC | Chaoyanggou | Granitic mylonite | 150 ± 1 | LA-ICP-MS |  |
| CF06-066 | NNCC | Molihaigou | Mylonite diorite | 128 ± 3 | SHRIMP |  |
| CF06-042 | NNCC | Chaoyanggou | Gneissic granite | 150 ± 1 | SHRIMP |  |
| CF06-066 | NNCC | Molihaigou | Gneissic granite | 128 ± 3 | SHRIMP |  |
| FW04-301 | NNCC | Gudaoling | Diorite enclave | 120 ± 1 | LA-ICP-MS |  |
| FW04-303 | NNCC | Gudaoling | Diorite enclave | 121 ± 2 | LA-ICP-MS |  |
| FW04-305 | NNCC | Gudaoling | Monzogranite | 121 ± 1 | LA-ICP-MS |  |
| FW04-315 | NNCC | Liangjiatai | Granite dyke | 128 ± 2 | LA-ICP-MS |  |
| FW04-319 | NNCC | Dafangshen | Quartz diorite | 124 ± 3 | LA-ICP-MS |  |
| FW04-337 | NNCC | Guanshui | Granodiorite | 131 ± 1 | LA-ICP-MS |  |
| JH-35 | NNCC | Gudaoling | Diorite enclave | 121 ± 3 | LA-ICP-MS |  |
| 03JH047 | NNCC | Haitangshan | Granite | 176 ± 1 | LA-ICP-MS |  |
| 03JH054 | NNCC | Haitangshan | Granite | 163 ± 1 | LA-ICP-MS |  |
| 03JH059 | NNCC | Haitangshan | Granite | 152 ± 1 | LA-ICP-MS |  |
| FW02-114 | NNCC | Jianchang | Quartz monzodiorite | 157 ± 1 | LA-ICP-MS |  |
| FW02-120 | NNCC | Jianchang | Granodiorite | 185 ± 2 | LA-ICP-MS |  |
| FW02-122 | NNCC | Jianchang | Granite | 190 ± 3 | LA-ICP-MS |  |
| FW02-135 | NNCC | Yangjiazhangzi | Monzogranite | 188 ± 2 | LA-ICP-MS |  |
| FW02-140 | NNCC | Yangjiazhangzi | Monzogranite | 189 ± 4 | LA-ICP-MS |  |
| FW02-144 | NNCC | Yangjiazhangzi | Monzogranite | 182 ± 2 | LA-ICP-MS |  |
| FW02-151 | NNCC | Jianlazi | Two-mica granite | 154 ± 2 | LA-ICP-MS |  |
| FW02-156 | NNCC | Jianlazi | Two-mica granite | 169 ± 10 | LA-ICP-MS |  |
| FW02-158 | NNCC | Jianlazi | Granite dyke | 182 ± 3 | LA-ICP-MS |  |
| FW02-168 | NNCC | Yiwulvshan | Two-mica granite | 163 ± 3 | LA-ICP-MS |  |
| FW02-170 | NNCC | Yiwulvshan | Granodiorite enclave | 153 ± 2 | LA-ICP-MS |  |
| FW02-176 | NNCC | Shishan | Monzogranite | 123 ± 3 | LA-ICP-MS |  |
| FW02-93 | NNCC | Kuangbang | Quartz monzodiorite | 182 ± 2 | LA-ICP-MS |  |
| FW02-95 | NNCC | Jianchang | Monzogranite | 153 ± 1 | LA-ICP-MS |  |
| CBL19 | NNCC | Chaobuleng | Bi-granite | 137 ± 2 | SHRIMP |  |
| Nianzigou | NNCC | Nianzigou | Bi-monzogranite | 152 ± 2 | SHRIMP |  |

118. Dai J, Mao J, Zhao C, Xie G, Yang F, Wang Y. New U–Pb and Re–Os age data and the geodynamic setting of the Xiaojiayingzi Mo (Fe) deposit, western Liaoning province, Northeastern China. Ore Geol Rev. 2009;35(2):235-44. doi: <https://doi.org/10.1016/j.oregeorev.2008.10.001>.

119. Deng JF, Mo XX, Zhao HL, Wu ZX, Luo ZH, Su SG. A new model for the dynamic evolution of Chinese lithosphere: ‘continental roots–plume tectonics’. Earth-Sci Rev. 2004;65(3):223-75. doi: <https://doi.org/10.1016/j.earscirev.2003.08.001>.

120. Fu L, Wei J, Kusky TM, Chen H, Tan J, Li Y, et al. The Cretaceous Duimiangou adakite-like intrusion from the Chifeng region, northern North China Craton: Crustal contamination of basaltic magma in an intracontinental extensional environment. Lithos. 2012;134-135:273-88. doi: <https://doi.org/10.1016/j.lithos.2012.01.007>.

121. Fu L, Wei J, Kusky TM, Chen H, Tan JUN, Li Y, et al. Triassic shoshonitic dykes from the northern North China craton: petrogenesis and geodynamic significance. Geol Mag. 2012;149(1):39-55. Epub 03/09. doi: 10.1017/S0016756811000173.

122. Guo L, Wang T, Castro A, Zhang JJ, Liu J, Li JB. Petrogenesis and evolution of late Mesozoic granitic magmatism in the Hohhot metamorphic core complex, Daqing Shan, North China. Int Geol Rev. 2012;54(16):1885-905.

123. Jiang N, Zhang S, Zhou W, Liu Y. Origin of a Mesozoic granite with A-type characteristics from the North China craton: highly fractionated from I-type magmas? Contrib Mineral Petr. 2009;158(1):113-30. doi: 10.1007/s00410-008-0373-2.

124. Li JY, Mo SG, He ZJ, Sun GH, Chen W. The timing of crustal sinistral strike-slip movementin the northern Great Khing'an ranges and its constrainton reconstruction of the crustal tectonic evolution ofNE China and adjacent areas since the Mesozoic. Earth Science Frontiers. 2004;172(3-4):223–49.

125. Miao L, Qiu Y, McNaughton N, Luo Z, Groves D, Zhai Y, et al. SHRIMP U–Pb zircon geochronology of granitoids from Dongping area, Hebei Province, China: constraints on tectonic evolution and geodynamic setting for gold metallogeny. Ore Geol Rev. 2002;19(3):187-204. doi: <https://doi.org/10.1016/S0169-1368(01)00041-5>.

126. Sun JF, Yang JH, Wu FY, Li XH, Yang YH, Xie LW, et al. Magma mixing controlling the origin of the Early Cretaceous Fangshan granitic pluton, North China Craton: In situ U–Pb age and Sr-, Nd-, Hf- and O-isotope evidence. Lithos. 2010;120(3):421-38. doi: <https://doi.org/10.1016/j.lithos.2010.09.002>.

127. Wu FY, Sun DY, Ge WC, Zhang YB, Grant ML, Wilde SA, et al. Geochronology of the Phanerozoic granitoids in northeastern China. J Asian Earth Sci. 2011;41(1):1–30.

128. Cai JH, Yan GH, Baolei MU, Kangxu R, Song B. Zircon U-Pb age, Sr-Nd-Pb isotopic compositions and trace element of Fangshan complex in Beijing and their petrogenesis significance. Acta Petrologica Sinica (in Chinese with English abstract). 2005;21(3):776-88.

129. Zeng QD, Liu JM, Zhang ZL, Qin F, Chen W, Zhang RB. Ore-forming time of the Jiguanshan porphyry molybdenum deposit, northern margin of North China Craton and the Indosinian mineralization. Acta Petrologica Sinica (in Chinese with English abstract). 2009;25(2):393-8.

130. Jiao ST, Yan DP, Zhang Q, Li CD, Wan B, Tian ZH. Zircon U-Pb age, geochemistry characteristics of Badaling granitoid complex and their geological significance. Acta Petrol Sin. 2013;29(3):769-80.

131. Xie HJ, Guang WU, Zhu MT. Geochronology and geochemistry of the Daolanghuduge A-type granite in Inner Mongolia,and its geological significance. Acta Petrologica Sinica (in Chinese with English abstract). 2012;28(2):483-94.

132. Qiang LI, Meng XY, Feng WU, Yang FQ, Liu F, Zhang ZX. LA-ICP-MS zircon U-Pb dating of intrusive rocks and its metallogenic significance in Sibozi-Liubozi molybdenum-copper deposit of Qinglong County,Hebei Province. Mineral Deposits (in Chinese with English abstract). 2012.

133. Liu C, Deng JF, Su SG, Xiao QH, Luo ZH, Wang QH, et al. Zircon SHRIMP dating of Yunmengshan gneissic granite and its geological significance. Acta Petrologica Et Mineralogica. 2004;23(2):141-6.

134. Liu Y, Ni ZY, Zhai MG, Shi YR, Yan G, Lu JS. Zircon SHRIMP U-Pb dating of granite in Chicheng county, northern Hebei Province and its geological implications. Journal of Mineralogy & Petrology. 2010;30(2):38-44.

135. Luo ZK, Guan K, Qiu YS, Miao LC, Qiu YM, McNaughton NJ, et al. Zircon SHRIMP U-Pb dating of Albite Dyke in Jinchangyu gold mine, Jidong Area, Hebei, China. Conributions to Geology & Mineral Resources Research. 2001;16(4):226-31 (in Chinese with English abstract).

136. Luo ZK, Qiu YS, Kang G, Miao LC, YMQiu, NJMcNaughton, et al. SHRIMP U-Pb Dating on Zircon from Yu’erya and Niuxinshan Granite Intrusions in Eastern Hebei Provence. Bulletin of Mineralogy Petrology & Geochemistry. 2001;20(4):286-8 (in Chinese with English abstract).

137. Luo ZK, Miao LC, Guan K, Qiu YS, Qiu YM, Mcnaughton NJ, et al. SHRIMP U-Pb zircon age of magmatic rock in Paishanlou gold mine district, Fuxin, Liaoning Province, China. Geochimica. 2001;30(5):483-90 (in Chinese with English abstract).

138. Miao LC, Qiu YM, Guan K, McNaughton N, Qiu YS, Luo ZK, et al. SHRIMP chronological study of the granitoids and mineralization in the Hadamengou gold deposit, Inner Mongolia. Mineral Deposits. 2000;19(2):182-90.

139. Wang HB, Liu GX, Xing CX. Zircon U-Pb age and geological implications of the molybdenum granites in Luogu, Mohe country, Heilongjiang province. Geology Resource 2010;19(2):186-90 (in Chinese with English abstract).

140. Wang YB, Han J, Li JB, Ou Yang ZX, Tong, Y., , Hou KJ. Age, petrogenesis and geological significance of die deformed granitoids in the Louzidian metamorphic core complex, southern Chifeng, Inner Mongolia; Evidence from zircon U -Pb dates and Hf isotopes [J]. Acta Petrologica Et Mineralogica (in Chinese with English abstract). 2010;29(6):763-78.

141. Wang ZL, Jin J, Li ZL, Lu BZ, Zhang ZY, Ke XS, et al. Zircon U-Pb ages and Hf isotopic characteristics of mineralized porphyries in the Mordaoga area,northern-central Da Hinggan Mountains,and their metallogenic significance. Acta Petrologica Et Mineralogica. 2010;29(6):796-810 (in Chinese with English abstract).

142. Wu FY, Lin JQ, Wilde SA, Zhang XO, Yang JH. Nature and significance of the Early Cretaceous giant igneous event in eastern China. Earth Pl Sc Lett. 2005;233(1):103-19. doi: <https://doi.org/10.1016/j.epsl.2005.02.019>.

143. Wu FY, Yang JH, Wilde SA, Zhang XO. Geochronology, petrogenesis and tectonic implications of Jurassic granites in the Liaodong Peninsula, NE China. Chem Geol. 2005;221(1):127-56. doi: <https://doi.org/10.1016/j.chemgeo.2005.04.010>.
